# Lithocholic Acid’s Ionic Compounds as Promising Antitumor Agents: Synthesis and Evaluation of the Production of Reactive Oxygen Species (ROS) in Mitochondria

**DOI:** 10.3390/antiox13121448

**Published:** 2024-11-25

**Authors:** Nuri M. Chobanov, Lilya U. Dzhemileva, Usein M. Dzhemilev, Vladimir A. D’yakonov

**Affiliations:** N.D. Zelinsky Institute of Organic Chemistry, Russian Academy of Sciences, Leninsky Prospect 47, Moscow 119991, Russia

**Keywords:** lithocholic acids, ionic compounds, dissipation of mitochondrial membranes, cytotoxicity, anticancer activity, apoptosis, cell cycle

## Abstract

The development of a methodology for the synthesis of new compounds with antitumor activity represents a significant and priority task within the field of medicinal chemistry. As a continuation of our research group’s earlier studies on the antitumor activity of ionic derivatives of natural compounds, we have synthesized a series of previously undescribed pyrazole ionic compounds through a series of transformations of lithocholic acid methyl ester. To investigate the biological activity of the newly synthesized lithocholic acid derivatives, a series of modern flow cytometry techniques were employed to assess their cytotoxic activity, effects on the cell cycle, and induction of apoptosis. This included the analysis of alterations in the mitochondrial potential, accumulation of ROS ions in mitochondria, and loss of cytochrome c. These compounds demonstrate promising antitumor activity through their effects on mitochondrial oxidation and phosphorylation processes. These compounds, which we have designated as “soft dissociators”, exhibit enhanced biopharmacological properties relative to the original lithocholic acid molecule.

## 1. Introduction

Bile acids possess distinctive characteristics that render them appealing targets for synthetic transformations. These include a chiral and rigid skeleton, as well as the presence of free carboxyl and hydroxyl groups [1].

Lithocholic acid (LA), a secondary bile acid, is formed by the bacterial 7-dehydroxylation of chenodeoxycholic acid (the primary bile acid) and ursodeoxycholic acid (the secondary bile acid). All of these processes occur within the gastrointestinal tract and are facilitated by the action of intestinal microflora. Lithocholic acid represents a rare example of a toxic endobiotic, and vertebrates have evolved a range of adaptive mechanisms to effectively detoxify it [2].

Furthermore, LA and its derivatives have been demonstrated to possess a range of biological activities. Many of these compounds serve as proteasome regulators [3,4], activate the vitamin D receptor, and enhance the interaction between cholecalciferols and the receptor [5,6], exhibit inhibitory activity against DNA polymerases β (pol β) [7], and have antibacterial [8] and antitumor effects [9,10,11,12].

It has been demonstrated that yeast cells cultivated in a culture medium comprising 0.2% glucose and lithocholic acid and fixed at varying stages of their chronological life cycle exhibit an altered age chronology for vital mitochondrial processes, including mitochondrial respiration, maintenance of electrochemical potential in the inner mitochondrial membrane, and establishment of stable levels of intracellular reactive oxygen species (ROS). These ROS are known to be produced primarily as by-products of mitochondrial respiration [13]. In other words, the results obtained imply that the addition of lithocholic acid contributes to the harmonization of the production of ROS ions, which can slow down the chronological aging of yeast. This is dependent on maintaining a certain “optimal”, non-toxic level of ROS ions in the cells [13,14]. This optimal, sublethal level of mitochondria-derived ROS ions is insufficient to cause significant oxidative damage to cellular components. However, it has been demonstrated to stimulate hormesis in response to stress by activating a complex signaling network that specifically alters epigenetic patterns and gene expression, thereby promoting longevity [15,16]. The use of lithocholic acid in the design of hybrid molecules (HMs) and ionic compounds (ICs) allows for the creation of hybrid molecules and ionic compounds with the potential ability to penetrate the mitochondrial membrane. Secondly, it allows for the synthesis of compounds with specified properties, namely, with antitumor pharmacophores. Thirdly, it allows for the obtaining of molecular hybrids with unique biological properties, which are potential “soft dissociators” of mitochondrial potential, affecting redox processes in mitochondria.

## 2. Materials and Methods

### 2.1. Chemistry

All commercial reagents were purchased from Sigma-Aldrich, St. Louis, MO, USA and Acros organics, Geel, Belgium. All commercially available solvents and reagents used were of analytical grade and without further purification. Reactions were monitored by TLC on Sorbfil plates. Column chromatography was carried out on Acrus silica gel (0.060–0.200 mm). ^1^H and ^13^C NMR spectra were obtained using a Bruker AM-300 spectrometer (Bruker, Leipzig, Germany) in CDCl_3_ operating at 300.13 MHz for ^1^H and 75.47 MHz for ^13^C and a Bruker AV 600 spectrometer in CDCl_3_ operating at 600.1 MHz for ^1^H and 150.9 MHz for ^13^C. High-resolution mass spectra with electrospray ionization were measured with a Bruker MicroOTOF II instrument (Bruker, Leipzig, Germany).

### 2.2. Cell Culturing

The human cancer cell lines Jurkat, K562, A549, and HEK293 were obtained from the European Authenticated Cell Culture Collection and subsequently cultured in accordance with established standard protocols and sterile techniques. The cells were maintained in RPMI 1640 (for Jurkat and K562) and DMEM (for A549 and HEK293) media (Gibco, Grand Island, NY, USA), supplemented with 4 μM glutamine, 10% FBS (Sigma, Kawasaki, Japan), and 100 units/mL penicillin-streptomycin (Sigma). All cell types were cultured in a humidified atmosphere of 5% CO_2_ at 37 °C. Subculturing was conducted at two- to three-day intervals. Subsequently, the cells were seeded into 24-well plates at a density of 5 × 10^4^ cells per well and incubated overnight. The cells were subcultured at two-day intervals at a seeding density of 1 × 10^5^ cells per 24-well plate in RPMI medium with 10% FBS.

#### 2.2.1. Preparation of Effluents and Solutions of Test Compounds for Biological Testing

The dissolution of the tested compounds was initially conducted in dimethyl sulfoxide (DMSO) at a stock solution of 100 mM in 10% DMSO. Thereafter, the solution was diluted in complete culture medium, specifically either Dulbecco’s Modified Eagle’s Medium (DMEM) or Roswell Park Memorial Institute (RPMI) medium. Substances were added at concentrations of 10, 1, 100, 10, 1, and 0.1 μM on the day after seeding and incubated for 24 h. Thereafter, the cells were washed with a PBS solution and stained with a 7-AAD dye according to the manufacturer’s protocol for flow cytometry. The CC50 value, which characterizes the cytotoxicity parameters (i.e., the concentration of the compound required for 50% inhibition of cell viability in vitro), was calculated, a logC vs. % inhibition plot was generated, and the data were statistically processed using Excel and GraphPad Prism v.8.0.2 (San Diego, CA, USA, 2019). The data obtained from three independent experiments were expressed as the mean of the three measurements for each concentration ± standard deviation, relative to the values of the control (0.1% DMSO), which were taken as 100%.

#### 2.2.2. Cytotoxicity Assay

The viability of the cells was evaluated through the use of a 7-AAD (7-aminoactinomycin D) dye (eBioscience™, San Diego, CA, USA). Following incubation with the test compounds, the cells were harvested, washed with phosphate-salt buffer (PBS), and centrifuged at 400× *g* for five minutes. The cell precipitates were resuspended in 200 μL of staining buffer for flow cytometry (PBS without calcium and magnesium, 2.5% FBS) and stained with a 1-mM 7-AAD dye solution for 15 min in the dark at room temperature. Subsequently, all experimental and control samples were analyzed on a NovoCyte Penteon flow cytometer (Agilent, Santa Clara, CA, USA).

#### 2.2.3. Apoptosis Assay

The quantification of apoptosis was achieved through the detection of phosphatidylserine, which is expressed on the outer surface of the membrane. This was accomplished by utilizing the Alexa Fluor^®^ 488 Annexin V fluorescent staining kit, which is designed for the identification of apoptotic and necrotic cells (ThermoFisher, Waltham, MA, USA). The Jurkat cells were incubated for 24 h with the tested compounds, then washed twice with cold PBS buffer. They were then resuspended in 100 μL of Annexin V binding buffer (10 mM HEPES, 140 mM NaCl, 2.5 mM CaCl_2_; pH 7.4). Annexin V (5 μL) and 7-AAD (1 μL) solutions were subsequently added to the reaction mixture and incubated for 15 min at room temperature in the dark. Subsequently, the stained cells were analyzed using the NovoCyte Penteon flow cytometer system (Agilent, USA).

#### 2.2.4. Cell Cycle Analysis

The cell cycle was analyzed using the MAK344 cell cycle assay kit (Sigma-Aldrich). Following a 24-h incubation period with the tested compounds, the cells were harvested, washed twice with phosphate-salt buffer (PBS), and centrifuged at 450× *g* for five minutes. The cell precipitate was resuspended and fixed with ice-cold 70% ethanol for a period of 24 h at a temperature of 0 degrees Celsius. Prior to staining, the cells were washed with PBS and incubated for 30 min with 0.5 mL of the cell cycle assay kit working solution at room temperature in the dark. The samples were subsequently analyzed using the NovoCyte Penteon flow cytometer system (Agilent, USA).

#### 2.2.5. ROS Ions Assay

The FlowCellect™ MitoStress Kit (Sigma-Aldrich, St. Louis, MO, USA) enables the concurrent assessment of two crucial cellular health indicators: mitochondrial superoxide generation, as gauged by the membrane-permeable dye MitoSOX Red, and phosphatidylserine expression on the surface of apoptotic cells, as evaluated by Annexin V binding in a single cell sample. The simultaneous measurement of these parameters has the additional benefit of reducing the time and number of samples required for analysis, while also enhancing the accuracy of the measurements obtained. A multiparametric evaluation of these cellular state markers permits the correlation and relationship of oxidative stress with apoptosis to be established. The assay is a valuable tool for screening compounds and elucidating the kinetics of dose-dependent processes.

#### 2.2.6. Mitopotential Assay

In our study, we conducted a cytometric assay that permitted the multiparametric evaluation of three markers of cellular condition: alterations in mitochondrial membrane potential (indicative of early apoptosis and cellular stress) and membrane permeabilization (a marker of cell death). The use of Millipore’s FlowCellect™ MitoPotential Red Kit reagents enables researchers to obtain information on the mitochondrial state and cell death in a relatively straightforward assay. The cells were treated with the synthesized compounds at a concentration of CC50 and incubated (37 °C, 5% CO_2_) for 6 h. Following this period, the cells were dissociated with Accutase solution, stained, and analyzed by flow cytometry (NovoCyte Flow Cytometry™ (Agilent, Santa Clara, CA, USA)) in accordance with the manufacturer’s protocols. The FlowCellect™ MitoPotential Red Kit was used (Merck, Darmstadt, Germany).

#### 2.2.7. Cytochrome c Assay

The quantification of cytochrome c release from mitochondria in apoptotic cells was employed to detect the mitochondrial pathway of apoptosis in cells using flow cytometry. The study employed a direct labeling approach utilizing the Anti-Cytochrome cFITC antibody, an Anti-IgG1-FITC isotype control, and optimized fixation, permeabilization and blocking buffers to detect cytochrome c by flow cytometry (Luminex^®^, Austin, TX, USA). The fluorescence of cytochrome c is higher in living cells, whereas lower levels are characteristic of apoptotic cells, in which cytochrome c is released from the mitochondria into the cytoplasm. Jurkat cells were incubated for four hours with the test substances at concentrations corresponding to their 24-h CC50 in these cells, after which they were analyzed by flow cytometry (NovoCyte Flow Cytometry™). The data were processed using the NovoExpress^®^ software (version 1.6.2) (ACEA (Agilent, Santa Clara, CA, USA)).

#### 2.2.8. Statistica

The normality of the distribution of the results obtained was checked. Chi-square was used for this purpose. Data were expressed as mean ± standard deviation. Student’s *t*-test was used for statistical comparisons of results. A *p*-value less than 0.01 and less than 0.05 was considered statistically significant. The regression analysis and stepwise analysis of variance (ANOVA) were used for statistical analysis.

## 3. Results

### 3.1. Chemistry

**Scheme 1:**
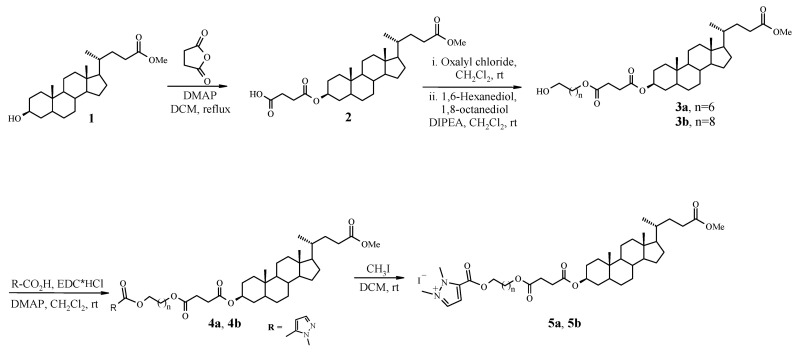
Synthesis of ionic compounds **5a**, **5b**.

The synthesis of the ionic compounds was accomplished in four steps from the methyl ester of lithocholic acid (**1**), which was previously synthesized from lithocholic acid according to a known procedure [17]. The first step involved the synthesis of the lithocholic acid derivative **2**, which was obtained through the interaction of ester **1** with succinic anhydride in the presence of DMAP in methylene chloride, under boiling conditions for 10 h (Scheme 1). The addition of 1,6-hexanediol and 1,8-octanediol to compound **2** was conducted via a sequential treatment of compound **1** with oxalyl chloride in dry methylene chloride. This was followed by stirring the reaction mass at room temperature for 12 h, dilution with a new portion of methylene chloride, and the addition of DIPEA and α,ω-diol. This resulted in the formation of derivatives **3a** and **3b**, respectively. The synthesis of imidazole derivatives of lithocholic acid (**4a**, **4b**) was achieved through the reaction of the 6-hydroxyhexyl derivative (**3a**) or the 8-hydroxyoctyl derivative (**3b**) with 1-methyl-1H-pyrazole-5-carboxylic acid in anhydrous CH_2_Cl_2_, in the presence of N-[3-(methylamino)propyl]-N’-ethylcarbodiimide hydrochloride (EDC*HCl) and 4-dimethylaminopyridine (DMAP).

The final step of the synthesis of target ionic compounds **5a**,**b** entailed the quantization of **4a**,**b**, which was carried out using methyliodide (MeI) in dry methylene chloride under stirring in an argon current at room temperature for 24 h (Scheme 1).

### 3.2. Biological Evaluation

#### 3.2.1. Cytotoxic Activity In Vitro

**Figure 1:**
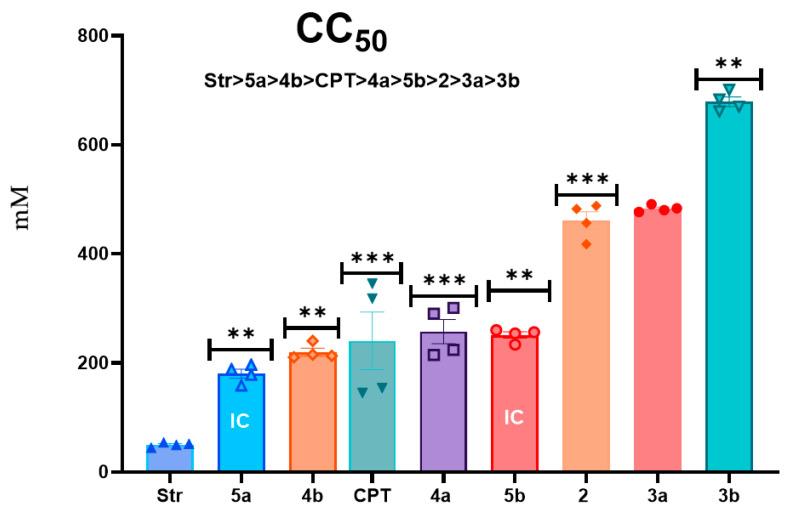
The cytotoxic activity (CC50) of the synthesized ionic compounds and lithocholic acid-based hybrid molecules in vitro was evaluated on cell cultures (Jurkat, K562, A549, and HEK293). The values depicted in the figure represent the mean for all four cell lines, while the individual values for each cell line are presented in Table S1. All synthesized compounds were injected into cell samples at a concentration corresponding to the CC50 value, the incubation period was 24 h. The presence of *** (*p* < 0.001), and ** (*p* < 0.01) indicates the reliability of the observed differences between the values obtained in the control sample and the samples treated with synthesized substances (the comparison was made by one-way ANOVA).

The biological activity of the synthesized compounds was evaluated using four cell lines: Jurkat, K562, A549, and HEK293. The data pertaining to the cytotoxicity of the aforementioned compounds are presented in Figure 1 and Table S1 of the Supplementary Information File. A comparison of the cytotoxicity parameters of each compound against a particular cell line revealed that Jurkat cells exhibited the greatest sensitivity to this library of compounds, while HEK293 cells demonstrated the highest resistance. Additionally, the selectivity of each compound against cell cultures did not exhibit significant differences, with the exception of the samples treated with staurosporine and compound **3a** HEK293 is a conditionally normal cell line that is frequently employed in the assessment of drug toxicity, xenobiotic toxicity, vaccine toxicity, and other related fields (Figure 1). Ionic compound **5a** was identified as the most toxic for all cell lines, exhibiting comparable cytotoxicity to staurosporine. However, unlike the latter, it demonstrated selective toxicity against the studied cell lines with high reliability (*p* < 0.05). Compound **5b** exhibited comparable cytotoxicity to camptothecin; however, it demonstrated a significantly inferior selective effect on cells. The least toxic compounds were **2, 3a**, and **3b**, with **2** and **3b** exhibiting a significant difference in the selective effect on cell lines. The obtained data are in good agreement with previous studies on the cytotoxicity of ionic compounds based on pentacyclic steroids [18]. Ionic compounds based on ursolic, betulinic, and oleanolic acids have been demonstrated to the greatest cytotoxicity against a range of cell lines in comparison to conventional hybrid molecules, namely, lithocholic acid derivatives. All compounds under investigation demonstrated CC50 values exceeding 100 mM in all cell lines utilized, suggesting that they did not elicit notable cytotoxicity but rather exhibited antiproliferative and anti-invasive properties [19]. It has been demonstrated that these molecules, in ionic form, exhibit enhanced permeability and solubility within biological membranes relative to their original, non-ionic counterparts [18].

#### 3.2.2. Study of Apoptosis Induction

In recent years, there has been a growing interest in investigating the biological effects of various ionic compounds on human cell lines. These include the colon carcinoma cell line Caco-2, the human T-lymphocytic leukemia cell line Jurkat, the lung carcinoma cell line A549, the human embryonic kidney cell line HEK 293T, and the promyelocytic leukemia cells HL-60. The focus of this research is the induction of apoptosis, or programmed cell death, in these cell lines [20,21,22,23,24]. Nevertheless, the impact of ionic compounds on the fundamental molecular processes underlying apoptosis remains poorly understood. The Jurkat cell line was selected for study due to its widespread use in apoptosis induction research [25,26].

**Figure 2:**
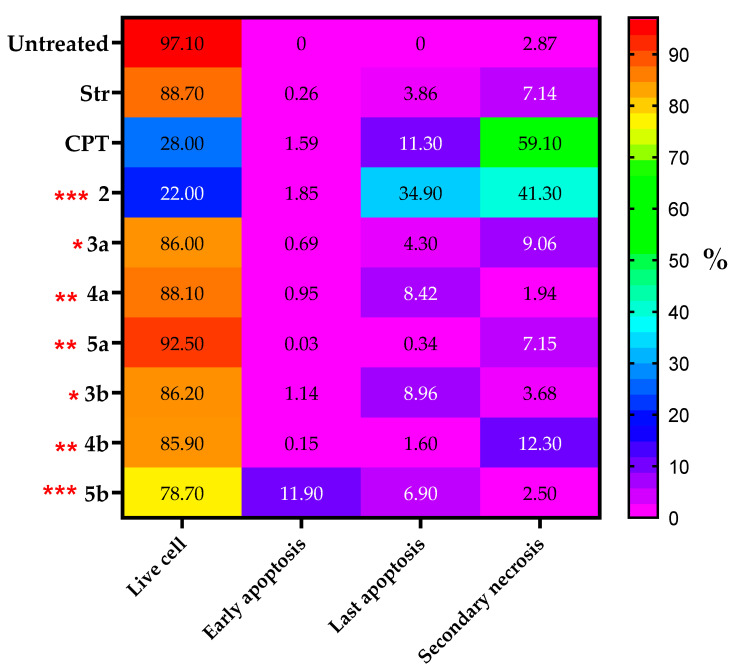
Graphical illustration of the ratio of apoptosis phases in Jurkat cells after exposure to ionic compounds and hybrid molecules based on lithocholic acid. All synthesized compounds were injected into cell samples at a concentration corresponding to the CC50 value, the incubation period was 24 h. The presence of *** (*p* < 0.001), ** (*p* < 0.01), and * (*p* < 0.05) indicates the reliability of the observed differences between the values obtained in the control sample and the samples treated with synthesized substances (the comparison was made by one-way ANOVA).

Figure 2 illustrates the stages of apoptosis in Jurkat culture cells. All synthesized compounds, including ionic compounds **5a** and **5b**, hybrid molecules, and the starting compounds for their synthesis, were incubated for 24 h with Jurkat cells (Figure 2). With regard to the induction of apoptosis in the Jurkat cell line, compound **2** exhibited properties most closely resembling those of camptothecin, and was comparable to it with respect to the evaluation of apoptotic phases. Therefore, camptothecin and compound **2** exhibited notable distinctions from the remaining compounds in the early, late apoptosis, and secondary necrosis phases (Figure 2). Furthermore, the ionic compounds **5a** and **5b** exhibited reliable differences between themselves with regard to the phases of apoptosis induction. Therefore, compound **5b** exhibits a more pronounced phase of early and late apoptosis, whereas compound **5a** displays a more pronounced secondary necrosis. In conclusion, it can be stated that ionic compounds **5a** and **5b** act less potently than camptothecin and compound **2,** resulting in weaker effects in the induction of apoptosis. This can be attributed to structural differences, namely, the unequal distance of the anion from the LCA molecule.

#### 3.2.3. Study of the Effect on the Cell Cycle

**Figure 3:**
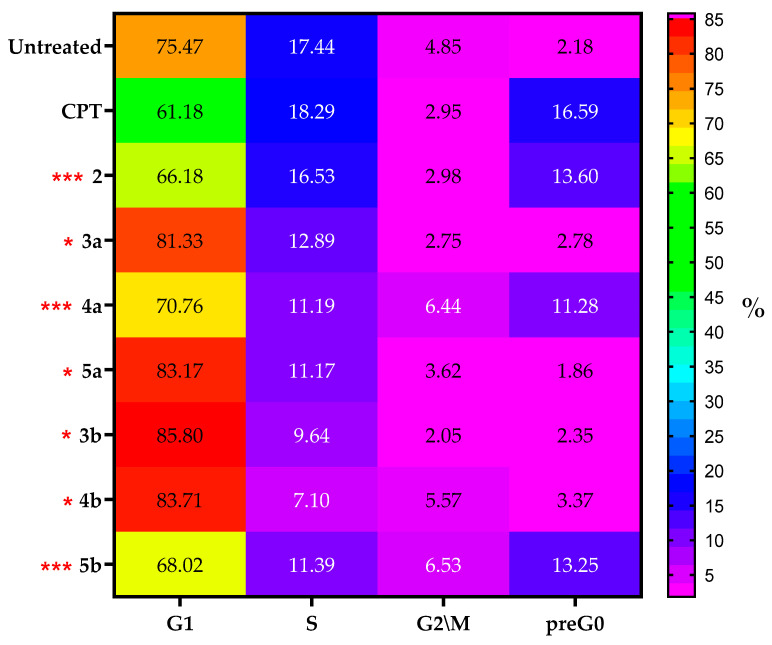
The ratio of cell cycle phases in Jurkat cells following exposure to lithocholic acid-based ionic compounds. All synthesized compounds were injected into cell samples at a concentration corresponding to the CC50 value, the incubation period was 24 h. The presence of *** (*p* < 0.001) and * (*p* < 0.05) indicates the reliability of the observed differences between the values obtained in the control sample and the samples treated with synthesized substances (the comparison was made by one-way ANOVA).

The impact of ionic compounds on the cell cycle is typically distributed across all phases, although certain phases are more susceptible to influence than others. Additionally, it was demonstrated that there is an inverse correlation between the length of the alkyl chain in the kathione of the ionic compound and the proportion of cells in the G1 phase. The impact of IS on the S phase is distinctive, frequently leading to an accumulation of cells in this phase and the inhibition of the G2/M phase [22]. A comparative analysis of the impact of the synthesized compounds on the cell cycle revealed that ionic compounds exhibited a more pronounced effect than hybrid molecules when all synthesized compounds were evaluated in conjunction with camptothecin. To illustrate, when the G1 phase was compared, the ionic compound **5b** co-compounded with camptothecin and compound **2** resulted in a reduction in the cell population to 68.02%, 61.18%, and 66.18%, respectively. In contrast, the administration of compound **5a** resulted in an increase in the G1 phase, reaching 83.17% compared to the control group (75.14%). The preG1 cell population was found to be affected by compound **5b** in a manner similar to camptothecin, resulting in an increase in this population up to 13.25%. In comparison, the control cells exhibited an index of 2.18% (Figure 3). Upon analysis of the S phase, it was observed that compound **2**, along with camptothecin, exhibited some degree of elongation, though this was not statistically significant when compared to the control. In contrast, ionic compounds **5a** and **5b**, along with hybrid compound **4a,** demonstrated a reduction in the cell population within the S phase (11.7%, 11.39%, and 11.19%, respectively). Therefore, both ionic compounds and lithocholic acid-based hybrid molecules impact all phases of the cell cycle, though they exert a particularly pronounced influence on the G1, preG1, and S phases (Figure 3).

#### 3.2.4. A Study of the Accumulation of Reactive Oxygen Species (ROS) in Mitochondria

**Figure 4:**
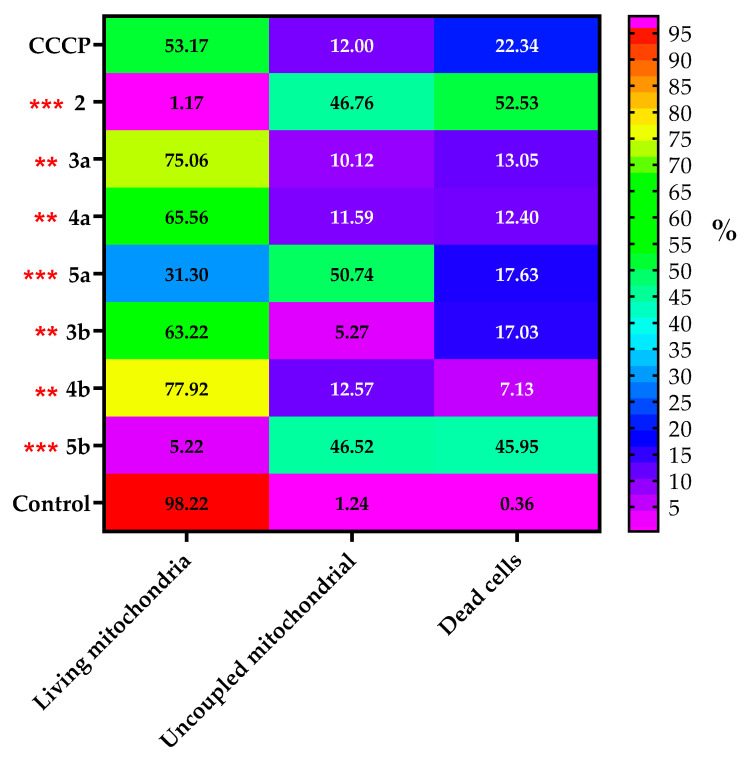
Simultaneous determination of the levels of cell death markers and mitochondrial membrane potential disruption in Jurkat cells exposed to a series of synthesized hybrid molecules and lithocholic acid-based ionic compounds. All synthesized compounds were injected into cell samples at a concentration corresponding to the CC50 value, the incubation period was 12 h. The presence of *** (*p* < 0.001), and ** (*p* < 0.01) indicates the reliability of the observed differences between the values obtained in the control sample and the samples treated with synthesized substances (the comparison was made by one-way ANOVA).

In order to gain a deeper comprehension of the impact of ionic compounds and hybrid molecules based on lithocholic acid, we conducted an in-depth analysis of the dissociation of the mitochondrial potential and the accumulation of ROS ions in mitochondria. This analysis served as an indicator of redox processes and the functioning of mitochondrial membranes. The mitochondrial potential is generated by the activity of electron-transport chain enzymes within the inner and outer membranes of the mitochondria. These processes facilitate the generation of ATP molecules within the mitochondria. During apoptosis, the collapse of the mitochondrial membrane potential (MMP) coincides with the opening of the mitochondrial permeability transition pore, which results in the release of cytochrome c and other enzymes into the cytosol. This, in turn, triggers subsequent events in the apoptotic cascade that can ultimately lead to cell death or, in a less severe form, dissociate the processes of oxidation and phosphorylation, dissipating energy into the surrounding space [27]. Figure 4 illustrates the cytometric detection of the effects of synthesized hybrid molecules and lithocholic acid-based ionic compounds on the mitochondrial membrane potential.

The loss of mitochondrial potential in Jurkat cells was determined using JC-1 dye in the presence of hybrid molecules and ionic compounds based on lithocholic acid. The dye JC-1 is a commonly utilized analytical tool in numerous research laboratories. Its fluorescence peak is detected on the APC channel in flow cytometry. JC-1 is a cationic, lipophilic dye that concentrates and forms reversible JC-10 red-fluorescent aggregates (λex = 540/λem = 590 nm) in the mitochondria of living cells with a polarized mitochondrial membrane. In cells undergoing apoptosis, the dissociation of the mitochondrial potential results in the inability of JC-1 to be retained within the mitochondria [28,29]. An additional dye, 7-AAD, is used to detect apoptosis caused by abnormalities in the mitochondrial membrane. It is also important to note that the characteristics of JC-1 staining may vary depending on the specific cell type and cell line under investigation [30]. In our investigation of mitochondrial potential dissociation, we employed the conventional oxidative phosphorylation dissociator, carbonyl cyanide m-chlorophenylhydrazone (CCCP), which is frequently utilized to induce the opening of transition pores and enhance mitochondrial membrane permeability to protons, ultimately leading to mitochondrial membrane potential dissipation (ΔΨm).

Thus, when the differences in mitochondrial membrane potential disruption were examined, the most significant differences were observed in compounds **3a** (*p* < 0.03), **4a** (*p* < 0.01), **4b** (*p* < 0.03), and **5b** (*p* < 0.01) when comparing these samples with control cells (Figure 4 and Figure S1 of the Supplementary Information File). In consideration of the chemical structure of the compounds, **3a**, **4a**, and **4b** are hybrid molecules, while **5b** is an ionic compound. All of the aforementioned compounds are derivatives of lithocholic acid. Given their impact on mitochondrial metabolism, and their capacity to disassociate the processes of oxidation and phosphorylation, influencing mitochondrial potential, it can be posited that they are of significance for cellular respiration. The authors posit that lithocholic acid induces substantial alterations in the mitochondrial membrane lipidome, which, in turn, give rise to notable modifications in mitochondrial morphology. These changes, in turn, influence the chronology of mitochondrial respiration, the electrochemical membrane potential, ATP synthesis, and ROS ions homeostasis [13].

The most notable breach of mitochondrial potential in cells subjected to treatment with compound **2** (the percentage of viable cells is 1.17%). It is probable that cell death occurs rapidly; therefore, the fixation of mitochondrial processes 12 h after exposure to the compound under study does not accurately reflect the dissociator’s effect, as the majority of cells are already dead by this time. The other compounds described above, including ionic compounds, have a milder and more prolonged effect, resulting in the survival of the majority of cells after 12 h of incubation.

Reactive oxygen species (ROS), including superoxide and peroxides, are continuously generated during cellular metabolic processes. The formation of ROS ions is typically counterbalanced by the action of antioxidant enzymes and other redox molecules. Nevertheless, an excess of ROS ions can result in cellular damage, manifested as damage to DNA, lipids, and proteins. Furthermore, this process has the potential to stimulate the formation of a range of free radicals and other oxidative radicals through the mitochondria, which can then lead to the activation of caspases and the induction of apoptosis. The dyes utilized in this experiment are MitoSOX™ and Annexin Alexa Fluor 488. The MitoSOX reagent is a cell-penetrating fluorogenic probe that is not fluorescent in its resting state. Upon the activation of oxidative processes, it emits a strong fluorescent signal in the PE channel of a flow cytometer. Furthermore, the dye is uniquely capable of localizing to mitochondria, rendering it an optimal choice for the detection of specific oxidative stress within these organelles. A comprehensive flow cytometry examination of diverse cellular populations typically uncovers robust MitoSOX fluorescence in cells that have undergone death. This phenomenon can be attributed to the release of the fluorophore from the mitochondria and the binding of MitoSOX Red to the nuclei of these cells. Accordingly, the experimental design employed a gating strategy to exclude dead cells from the analysis of superoxide generation by mitochondria via flow cytometry.

**Figure 5:**
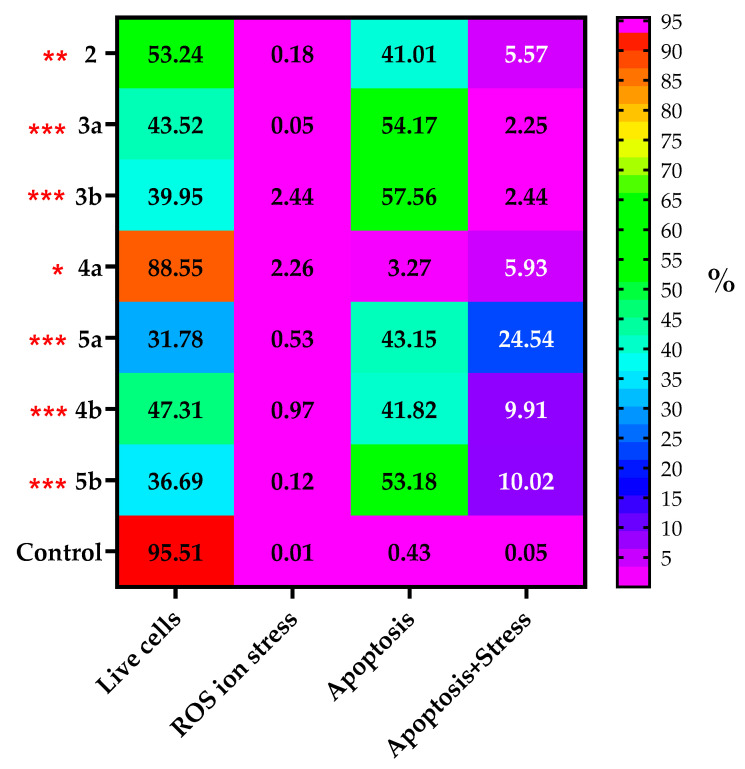
Effect of synthesized compounds on intracellular ROS levels and mitochondrial potential ΔΨm in Jurkat cells treated with hybrid molecules and ionic compounds. All synthesized compounds were injected into cell samples at a concentration corresponding to the CC50 value, the incubation period was 4 h. The presence of *** (*p* < 0.001), ** (*p* < 0.01), and * (*p* < 0.05) indicates the reliability of the observed differences between the values obtained in the control sample and the samples treated with synthesized substances (the comparison was made by one-way ANOVA).

In our research, we have presented a study of the ROS ions level when investigating the effect of hybrid molecules and ionic compounds to gain a deeper understanding of the processes occurring in the mitochondria of Jurkat cells, including the processes of oxidation and phosphorylation on mitochondrial membranes (Figure 5 and Figure S2 of the Supplementary Information File). Therefore, compounds **5a** and **5b** were identified as the compounds that elicited the highest production of ROS ions within the cellular environment. This finding is not in contradiction with the extensive literature on the subject and with our own research, which indicates that ionic compounds can induce mitochondrial apoptosis [18,22,23]. Concurrently, the proportion of cells stained with Annexin and MitoSOX in compounds **5a** and **5b** was also comparable, reaching 24.54% and 10.02%, respectively. This finding suggests that ionic compounds exert the most pronounced impact on mitochondria and substantiates their capacity to induce apoptosis via the mitochondrial pathway (Figure 5).

#### 3.2.5. A Study of the Release of Cytochrome c from Mitochondria

**Figure 6:**
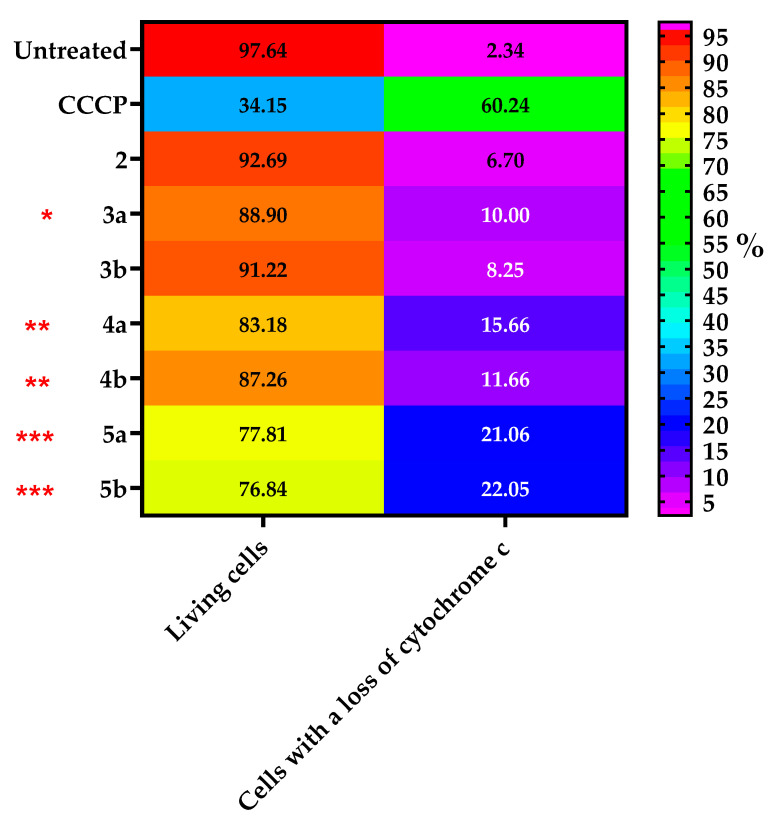
Cytochrome c detection in Jurkat culture cells stained with FlowCellect Cytochrome c Kit. The control compound, carbonyl cyanide m-chlorophenylhydrazone (CCCP), was administered at a concentration corresponding to its CC50 value. All synthesized compounds were injected into cell samples at a concentration corresponding to the CC50 value, the incubation period was 6 h. The presence of *** (*p* < 0.001), ** (*p* < 0.01), and * (*p* < 0.05) indicates the reliability of the observed differences between the values obtained in the control sample and the samples treated with synthesized substances (the comparison was made by one-way ANOVA).

Cytochrome c is a soluble protein that is localized in the intermembrane space of mitochondria. It functions as an electron carrier in the process of oxidative phosphorylation during respiration, transferring electrons from the cytochrome bc1 complex to cytochrome oxidase on the surface of the inner mitochondrial membrane. This plays a crucial role in the formation of the mitochondrial membrane potential difference. The release of cytochrome c and other pro-apoptotic proteins into the cytosol is a key event in the initiation of the intrinsic (mitochondrial) pathway of cell death, which is triggered by the induction of various types of apoptosis and related events, namely, mitochondrial depolarization and cardiolipin peroxidation. Upon release, cytochrome c interacts with other mitochondrial intermembrane proteins, such as procaspase 9 and Smac, as well as cytosol factors, including apoptotic protease-activating factor-1 and ATP, to activate a cascade of caspases that promote cell death. The quantification of cytochrome c release from the mitochondria of apoptotic cells can be employed to characterize the mitochondria-driven pathway of cell death [31]. Modern methods of cytochrome c release assessment include Western blotting, ELISA, and fluorescence microscopy. These methods have a number of disadvantages, including the fact that they are labor-intensive, make it difficult to examine several samples in parallel, and often require cell lysis, which entails a loss of information about cytochrome c translocation in mitochondria. Furthermore, in methods involving the fractionation and isolation of mitochondrial proteins, which often employ harsh mechanical means such as homogenization, incomplete or excessive mitochondrial disruption may occur, which may result in an over- or underestimation of the amount of cytochrome c in the cytoplasm. The utilization of methodologies for the staining of cytochrome c within the mitochondria in intact cells without rupture, followed by detection via flow cytometry, enables the most accurate detection of cytochrome c within the cell and the quantification of the percentage of cells exhibiting reduced or absent cytochrome c content within their mitochondria [32]. The present study demonstrates that the synthesized ionic compounds and hybrid molecules based on lithocholic acid induce the release of cytochrome c into the cytoplasm, thereby initiating mitochondrial apoptosis. The results of the flow cytometry analysis and the corresponding histogram are presented in Figure 6 and Figure S3 of the Supplementary Information File.

Two mitochondrial events are known to mediate apoptosis: the appearance of mitochondrial permeability with a decrease in membrane potential and the release of pro-apoptotic proteins from the mitochondrial intermembrane space. The translocation of pro-apoptotic proteins from the mitochondrial intermembrane space into the cytosol represents a further crucial event that ultimately results in the subsequent activation of caspases. The most extensively studied of these proteins is cytochrome c, which, upon translocation into the cytosol, forms a multimeric complex with Apaf-1, thereby activating caspases [31].

As illustrated in Figure 6 and Figure S3 (Supplementary Information File), all synthesized compounds exhibit cytochrome release from mitochondria, and all display reliable differences with the most prevalent and well-characterized mitochondrial respiratory chain uncoupler, carbonyl cyanide m-chlorophenylhydrazone. The attachment of lipophilic moieties to a variety of small molecules is a common method for the synthesis of mitochondria-targeted compounds with specific activity [33]. The majority of compounds synthesized in our research, including hybrid molecules and ionic compounds containing lithocholic acid, have an uncoupling effect and result in the loss of cytochrome c due to the uncoupling of oxidation and phosphorylation processes in mitochondria. When compared to carbonyl cyanide m-chlorophenylhydrazone, it can be observed that it causes a significantly greater dissipation of the mitochondrial membrane than compounds **3a**, **3b**, **4a**, **4b**, **5a,** and **5b**. A comparison of the ionic compounds (**5a** and **5b**) and hybrid molecules (**4a** and **4b**) reveals that the greatest loss of cytochrome c occurs as a result of the action of the ionic compounds. Therefore, the synthesized ionic compounds and hybrid molecules based on lithocholic acid are more “soft” dissociators than, for example, the well-known and widely used CCCP. These compounds, which exhibit a pronounced action as dissipators of mitochondrial potency and relatively low toxicity in comparison with staurosporine, may prove useful as antitumor and antibacterial agents.

## 4. Conclusions

The majority of redox processes occur within the mitochondria, where energy-intensive molecules are produced through the oxidation of major substrates such as glucose, pyruvate, and NADH. This oxidation creates a proton gradient across the inner mitochondrial membrane, establishing an electrochemical potential (ΔΨm) that is essential for the synthesis of adenosine triphosphate (ATP). The energy stored in ΔΨm is primarily utilized for ATP synthesis, otherwise known as oxidative phosphorylation. It is important to note that not all of the energy present in the electrochemical gradient is utilized for ATP synthesis. Some of the energy is expended in “proton leakage” reactions, whereby protons pumped out of the matrix are able to flow back up the proton gradient through proton-conducting pathways in the inner membrane that bypass ATP synthesis [34]. Consequently, the energy derived from the metabolic oxidation reaction is dissipated as heat. Proton leakage that is not productive, or mitochondrial uncoupling, is a physiologically significant process, accounting for approximately 20% of the rate of basal metabolism. Mitochondrial uncoupling is a crucial process not only for normal cells but also for the metabolic pathways involved in carcinogenesis. Firstly, the generation of free oxygen species is involved in the regulation of various physiological processes in cancer cells, and activates signaling pathways for cell growth and proliferation. However, when ROS ions are overproduced, they can be harmful to the cell by initiating cell death pathways. It can be concluded that the regulation of free oxygen species levels in cancer cells is of great importance for the physiology, growth, and survival of tumor cells. It is thought that mitochondrial uncoupling has a natural antioxidant effect that increases the respiration rate and thus likely attenuates the production of ROS ions [34]. It is well established that elevated levels of mitochondrial uncoupling are present in numerous chemoresistant cancer cell lines, a phenomenon that may serve to safeguard the survival of tumor cells. In this study, a series of novel hybrid molecules and ionic compounds based on lithocholic acid containing an imidazole moiety were synthesized. It has been demonstrated that ionic compounds and hybrid molecules based on lithocholic acid possess apoptosis-inducing properties, affect all phases of the cell cycle, dissociate mitochondrial potential, and induce ROS ions production. In comparison to the established mitochondrial potential dissociator, carbonyl cyanide m-chlorophenylhydrazone (CCCP), the synthesized hybrid molecules and ionic compounds demonstrate a less pronounced capacity to dissociate the mitochondrial potential and promote the generation of free oxygen species. In consideration of the aforementioned findings, it can be postulated that ionic compounds and hybrid molecules derived from lithocholic acid possess the capacity to regulate mitochondrial metabolism with relatively minimal effects.

## Data Availability

The data presented in this study are available in this article.

## Supplementary Materials

The following supporting information can be downloaded at: https://www.mdpi.com/article/10.3390/antiox13121448/s1, Figures S1–S3 and Tables S1 and S2. ^1^H and ^13^C NMR spectra of the synthesized compounds.
