# Supplementary material for: Lithocholic Acid’s Ionic Compounds as Promising Antitumor Agents: Synthesis and Evaluation of the Production of Reactive Oxygen Species (ROS) in Mitochondria
Source: Antioxidants (Basel). 2024 Nov 25;13(12):1448. doi: 10.3390/antiox13121448 (PMC11672617; doi:10.3390/antiox13121448)
Supplement: Supplementary file 1 [file antioxidants-13-01448-s001.zip › antioxidants-3292976-supplementary.pdf]

Supporting information for

Lithocholic Acid's Ionic Compounds as Promising Antitumor Agents: Synthesis and Evaluation of the Production of Reactive Oxygen Species (ROS) in Mitochondria

Nuri M. Chobanov, Lilya U. Dzhemileva *, Usein M. Dzhemilev,

and Vladimir A. D'yakonov *

N.D. Zelinsky Institute of Organic Chemistry, Russian Academy of Sciences,

Leninsky Prospect 47, Moscow 119991, Russia

** Correspondence: Dzhemilev@mail.ru (L.U.D.)*
DyakonovVA@gmail.com (V.A.D.);

Table of contents

1. ^{1}H and ^{13}C NMR and mass spectra of the compounds **2, 3a, 3b, 4a, 4b, 5a, 5b**.
2. Biological studies.

Experimental Section

General

All commercial reagents were purchased from Sigma-Aldrich and Acros organics. Betulinic acid was prepared from commercially-available betulin by a reported procedure [1]. All commercially-available solvents and reagents used were of analytical grade and without further purification. Reactions were monitored by TLC on Sorbfil plates. Column chromatography was carried out on Acrus silica gel (0.060–0.200 мм). Optical rotations were measured on a Perkin–Elmer 341 polarimeter. Melting points were recorded on Stuart SMP3. IR spectra were recorded on Bruker VERTEX 70V using KBr discs over the range of 400–4000 cm^{-1}. ^{1}H and ^{13}C NMR spectra were obtained using a Bruker Ascend 500 spectrometer in $CDCl_3$ operating at 500 MHz for ^{1}H and 125 MHz for ^{13}C and a Bruker AVANCE 400 spectrometer in $CDCl_3$ operating at 400 MHz for ^{1}H and 100 MHz for ^{13}C. High-resolution mass spectra with electrospray ionization were measured with a Bruker MicroOTOF II instrument; external calibration of the mass spectrometer was performed using the Electrospray Tuning mix (Agilent).

*General procedure for the synthesis of compound **2**.*

To a stirred solution of **1** (390.6 mg, 1 mmol) and DMAP (148.06 mg, 1.2 mmol) in dry CH_2Cl_2 (10 ml), succinic anhydride (400.97 mg, 4 mmol) was added at room temperature. The resulting reaction mixture was refluxed for 10 h. After completion of the reaction, the solvent was evaporated under reduced pressure. The residue was diluted with EtOAc (20 ml) and washed with water (2x20 ml) and brine (2x20 ml). The organic layer was dried over $MgSO_4$ and concentrated.

The crude product was purified by column chromatography (silica gel) using petroleum ether/EtOAc = 4/1 as the elution solvent to afford compound **2**.

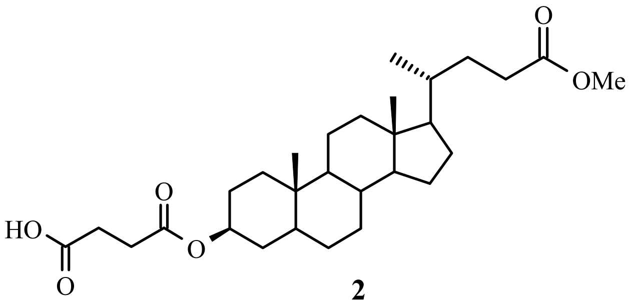

2

^{1}H NMR (300 MHz, Chloroform-*d*) δ 3.60 (s, 1H), 2.66 – 2.47 (m, 2H), 2.37 – 2.07 (m, 1H), 1.96 – 1.55 (m, 8H), 1.33 (s, 3H), 1.24 – 1.10 (m, 1H), 1.10 – 0.96 (m, 1H), 0.96 – 0.81 (m, 1H), 0.58 (s, 1H).

^{13}C NMR (75 MHz, Chloroform-*d*) δ 177.75, 174.92, 171.75, 75.06, 56.53, 56.05, 51.57, 42.81, 41.97, 40.49, 40.19, 35.86, 35.43, 35.07, 34.65, 32.23, 31.14, 31.08, 29.34, 29.04, 28.44, 28.25, 27.08, 26.64, 26.38, 24.25, 23.38, 20.91, 18.34, 12.10.

MS (ESI): 491.35 [M+H]. Calculated for $C_{29}H_{46}O_6$: 490.33.

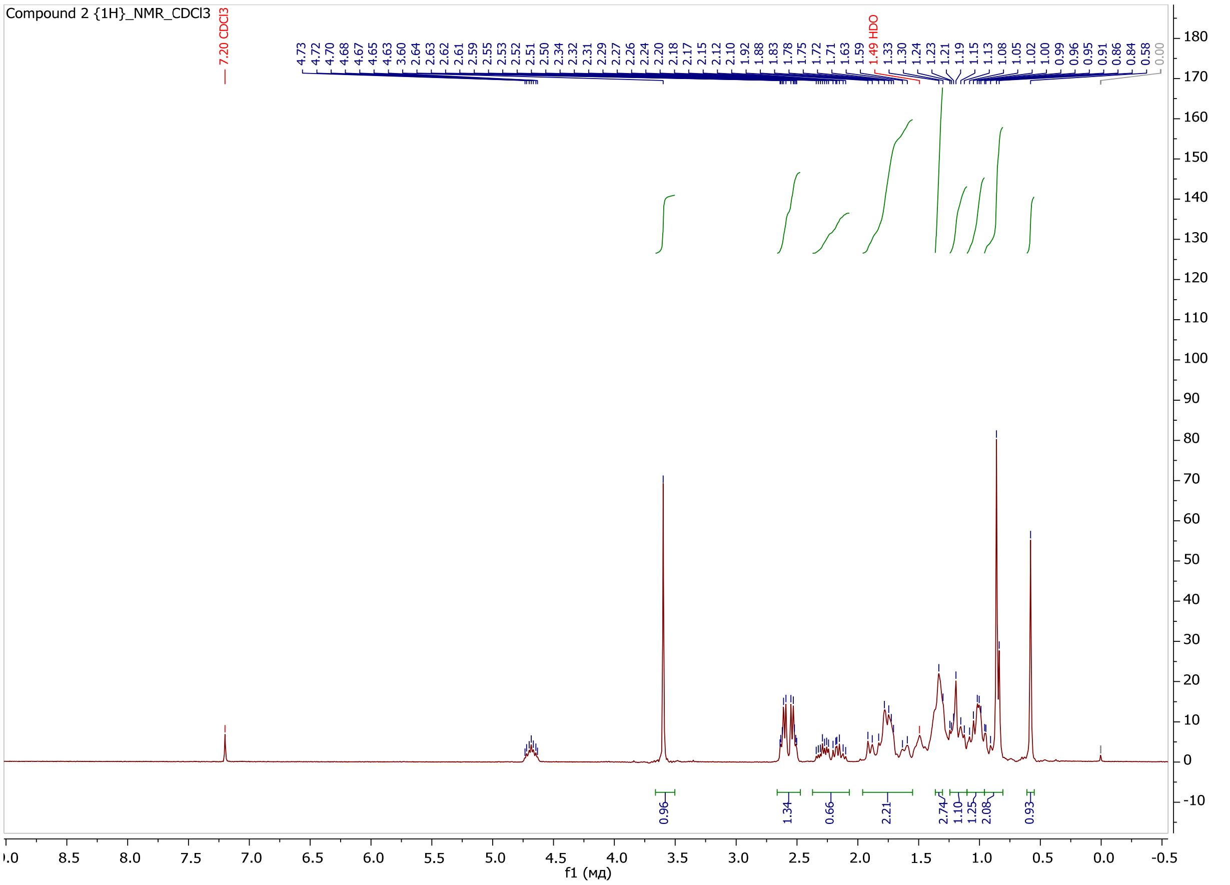

S1. ^{1}H NMR spectrum of compound **2**.

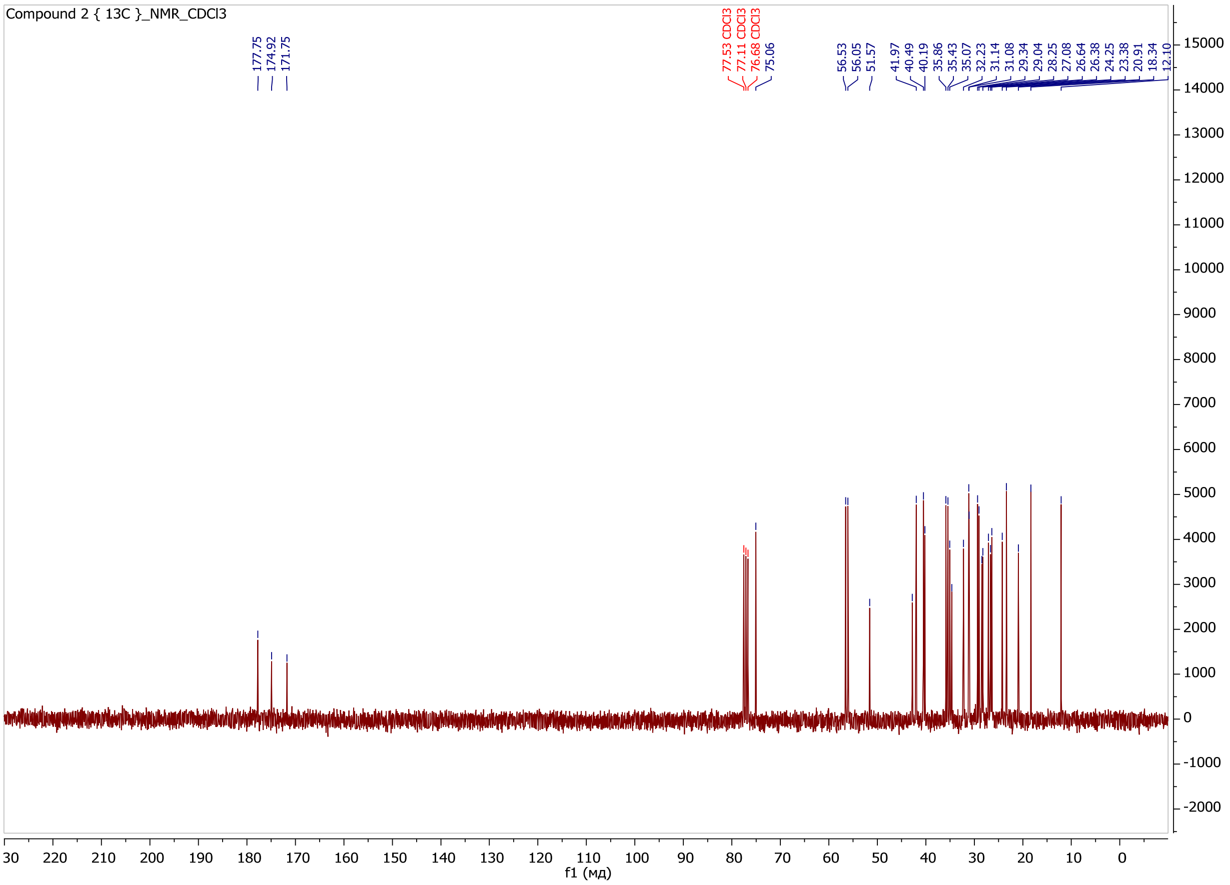

S1. ^{13}C NMR spectrum of compound **2**.

*General procedure for the synthesis of derivatives 3**a**, 3**b**.*

To a solution of 3α-((hydroxysuccinyl)oxy-5β-cholan-24-oate **2** (466 mg, 0.95 mmol) in anhydrous CH_2Cl_2 (20 mL) at 0 °C, oxalyl chloride (1.62 mL, 17.1 mmol) was added. After stirring at room temperature overnight, the mixture was evaporated, and co-evaporated with dry hexane (3 × 10 mL). The residue was dissolved in dry CH_2Cl_2 (20 mL), and then DIPEA (0.494 mL, 2.85 mmol) and 1,6-hexanediol (228 mg, 1.9 mmol) or 1,8-octanediol (277.4 mg, 1.9 mmol) were added at 0 °C. After stirring at room temperature for 24 h, the solvent was evaporated. The crude product was purified by column chromatography (silica gel) using petroleum ether/EtOAc = 1/1 as the elution solvent to afford lithocholic derivatives **3a, 3b**.

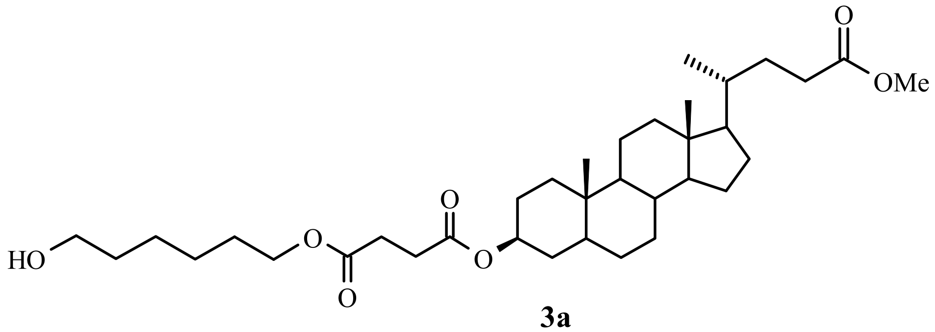

3a

6-Hydroxyhexyl 3α-(hydroxysuccinyl)oxy-5β-cholan-24-oate (***3a)***.

^{1}H NMR (500 MHz, Chloroform-*d*) δ 4.73 (t, *J* = 9.9, 4.9 Hz, 1H), 4.09 (t, *J* = 6.4 Hz, 2H), 3.66 (s, 3H), 3.64 (t, *J* = 6.5 Hz, 2H), 2.65 – 2.55 (m, 4H), 2.35 (t, *J* = 15.3, 10.2, 5.1 Hz, 1H), 2.28 – 2.17 (m, 1H), 2.00 – 1.22 (m, 21H), 1.16 (dd, *J* = 12.4, 3.5 Hz, 1H), 1.11 (d, *J* = 9.5 Hz, 1H), 1.10 – 0.97 (m, 5H), 0.92 (d, *J* = 2.4 Hz, 6H), 0.91 (s, 2H), 0.64 (s, 3H).

^{13}C NMR (126 MHz, Chloroform-*d*) δ 174.74, 172.41, 171.80, 77.26, 77.00, 76.75, 74.76, 64.66, 62.73, 56.47, 55.99, 51.44, 42.72, 41.88, 40.42, 40.12, 35.77, 35.34, 34.99, 34.57, 32.57, 32.19, 31.05, 30.99, 29.54, 29.25, 28.55, 28.16, 26.99, 26.59, 26.30, 25.67, 25.34, 24.16, 23.29, 20.82, 18.25, 12.01.

MS (ESI): 613.4075 [M+Na]. Calculated for $C_{35}H_{58}O_7Na$: 613.4080.

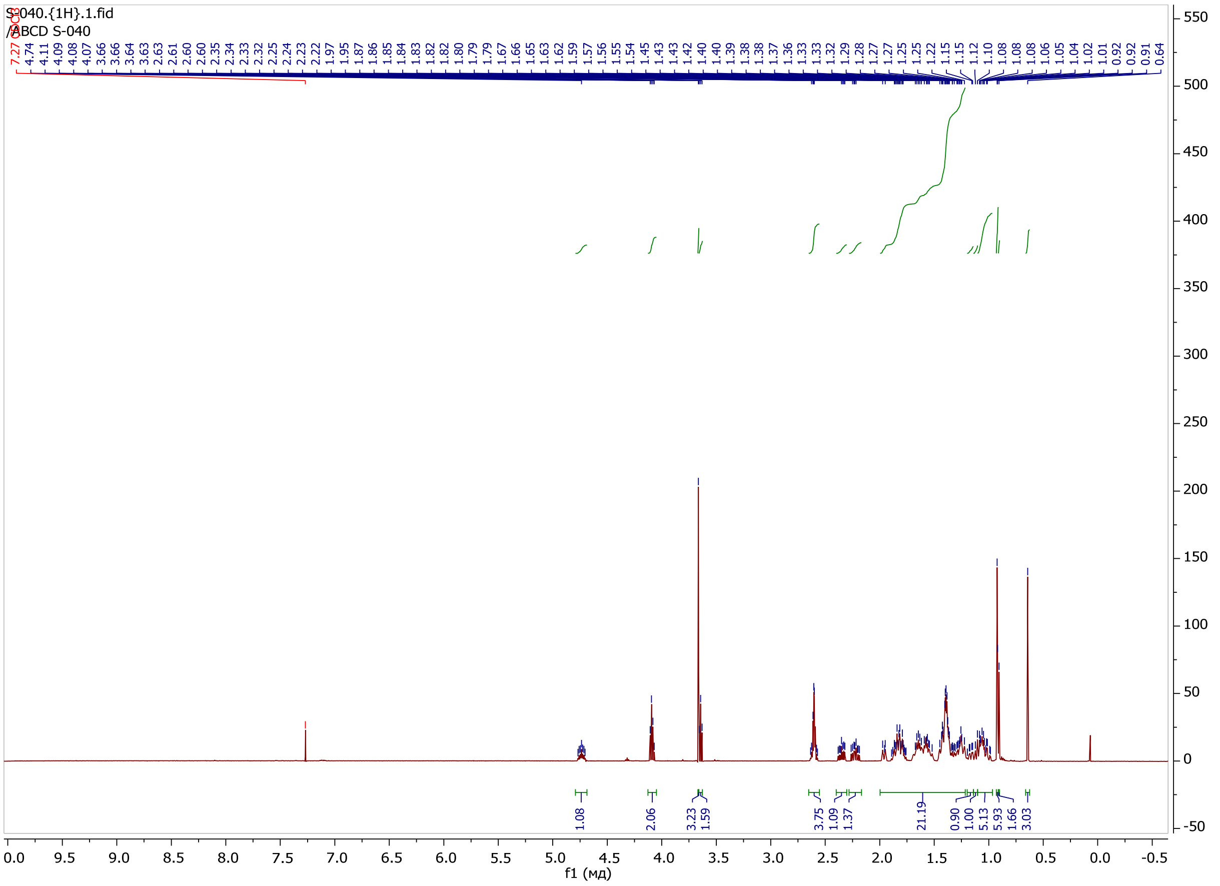

S2. ^{1}H NMR spectrum of compound **3a**.

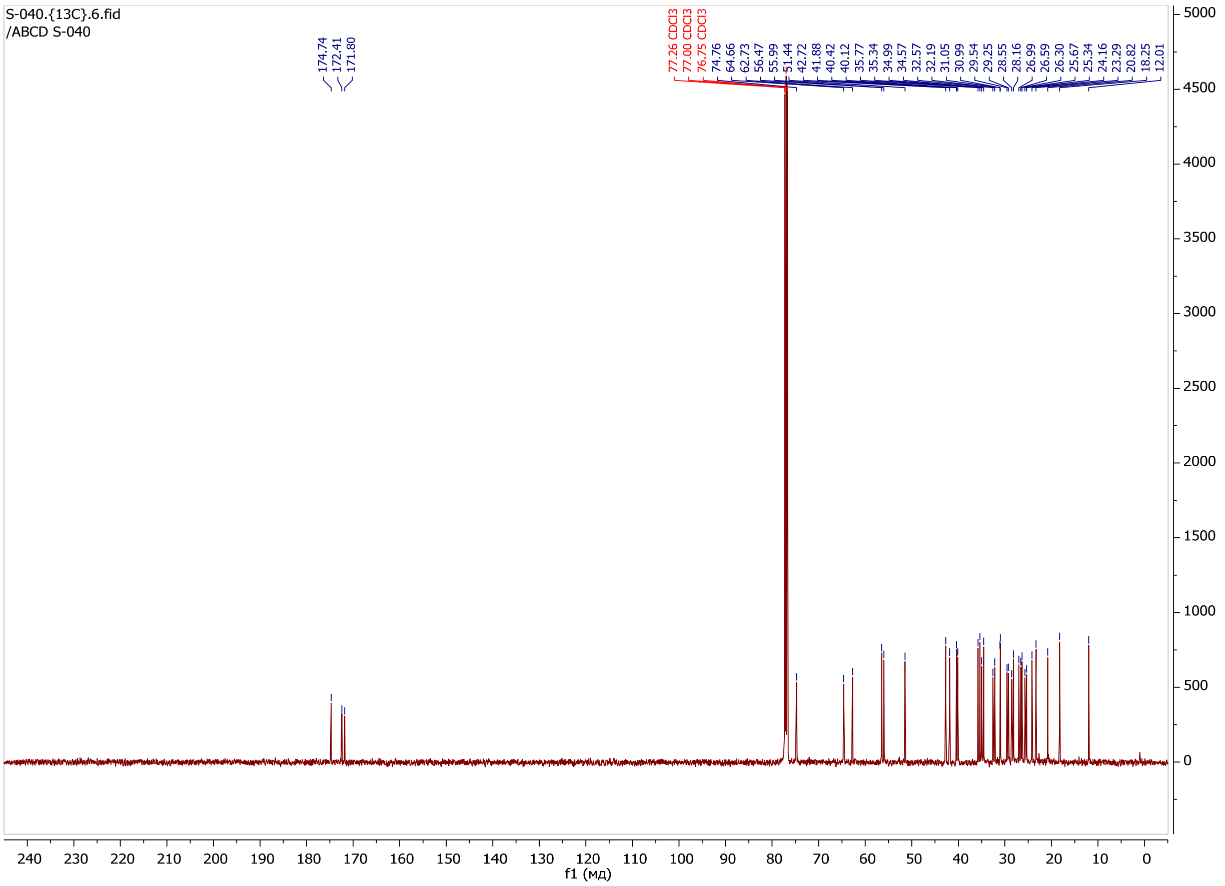

S2. ^{13}C NMR spectrum of compound **3a**.

Display Report

Analysis Info

Analysis Name	D:\Data\Kolotyrkina\2024\Chobanov\1111011.d	Acquisition Date	11.11.2024 12:57:00
Method	tune_low.m	Operator	BDAL@DE
Sample Name	/ABCD S-40	Instrument / Ser#	micrOTOF 10248
Comment	C35H58O7 clb added CH3OH		

Acquisition Parameter

Source Type	ESI	Ion Polarity	Positive	Set Nebulizer	0.4 Bar
Focus	Not active			Set Dry Heater	180 °C
Scan Begin	50 m/z	Set Capillary	4500 V	Set Dry Gas	4.0 l/min
Scan End	3000 m/z	Set End Plate Offset	-500 V	Set Divert Valve	Waste

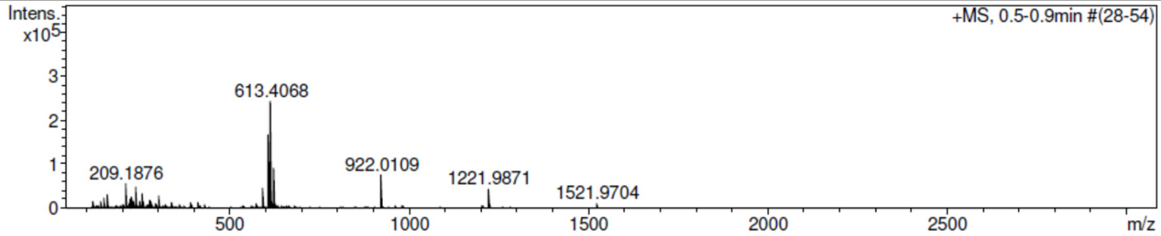

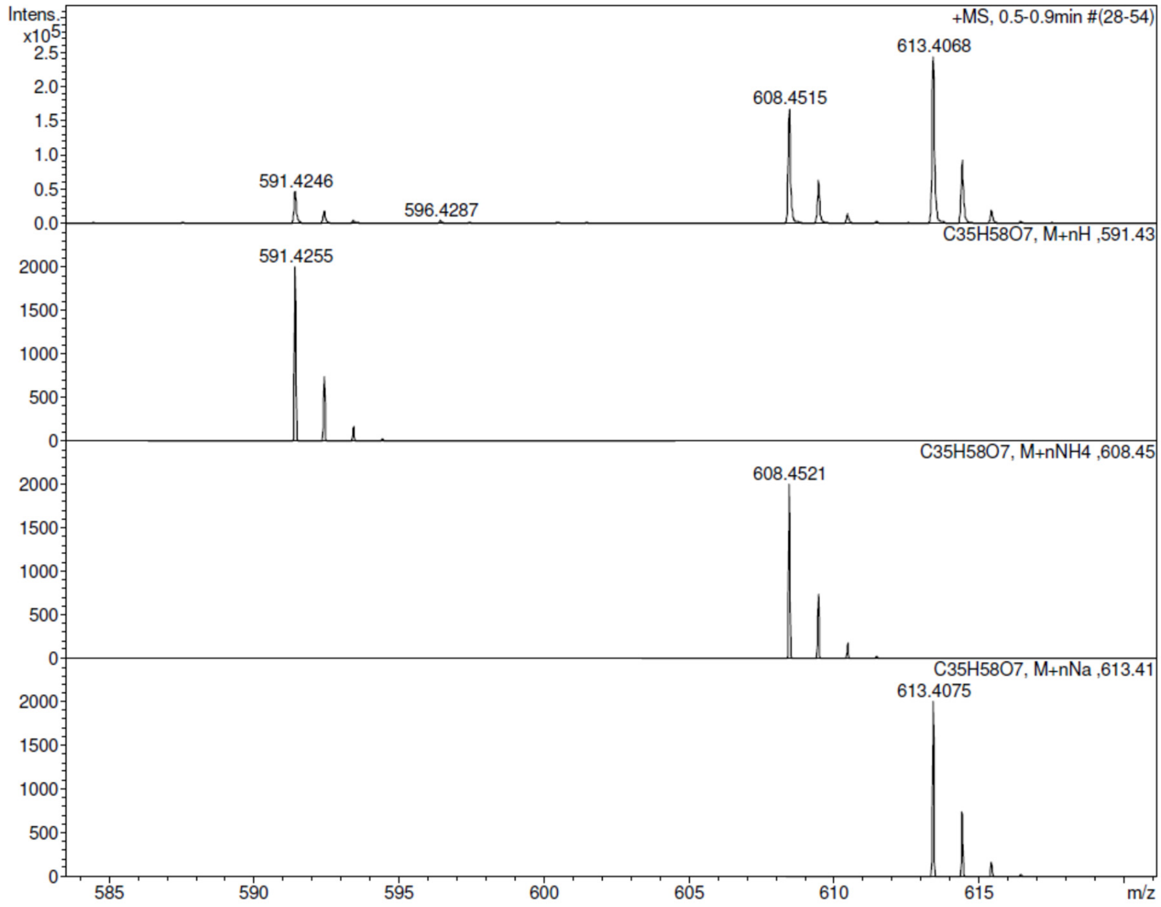

S2. Mass spectrum of compound **3a**.

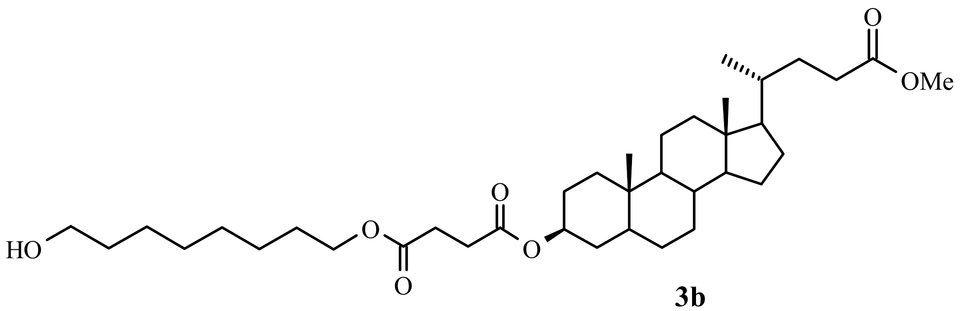

3b

8-Hydroxyoctyl 3α-(hydroxysuccinyl)oxy-5β-cholan-24-oate ***(3b).***

^{1}H NMR (600 MHz, DMSO-d_6) δ 3.95 (t, J = 6.7 Hz, 1H), 3.44 – 3.39 (m, 6H), 2.53 – 2.43 (m, 4H), 2.28 – 2.20 (m, 1H), 2.17 – 2.07 (m, 1H), 1.87 (d, J = 12.1 Hz, 1H), 1.80 – 1.63 (m, 4H), 1.57 – 1.46 (m, 1H), 1.45 – 1.37 (m, 10H), 1.35 – 1.19 (m, 18H), 1.19 – 0.95 (m, 6H), 0.95 – 0.88 (m, 1H), 0.85 – 0.80 (m, 5H), 0.55 (s, 3H).

^{13}C NMR (151 MHz, DMSO-d_6) δ 174.45, 172.24, 171.62, 74.51, 64.65, 64.59, 61.87, 61.84, 56.36, 55.88, 51.40, 41.81, 40.34, 40.01, 35.69, 35.23, 34.96, 32.81, 32.79, 32.14, 30.94, 30.92, 29.47, 29.43, 29.30, 29.19, 28.52, 28.12, 26.98, 26.54, 26.27, 25.81, 24.13, 23.35, 20.77, 18.29, 12.06.

MS (ESI): 641.4396 [M+Na]. Calculated for $C_{37}H_{62}O_7Na$: 641.4393.

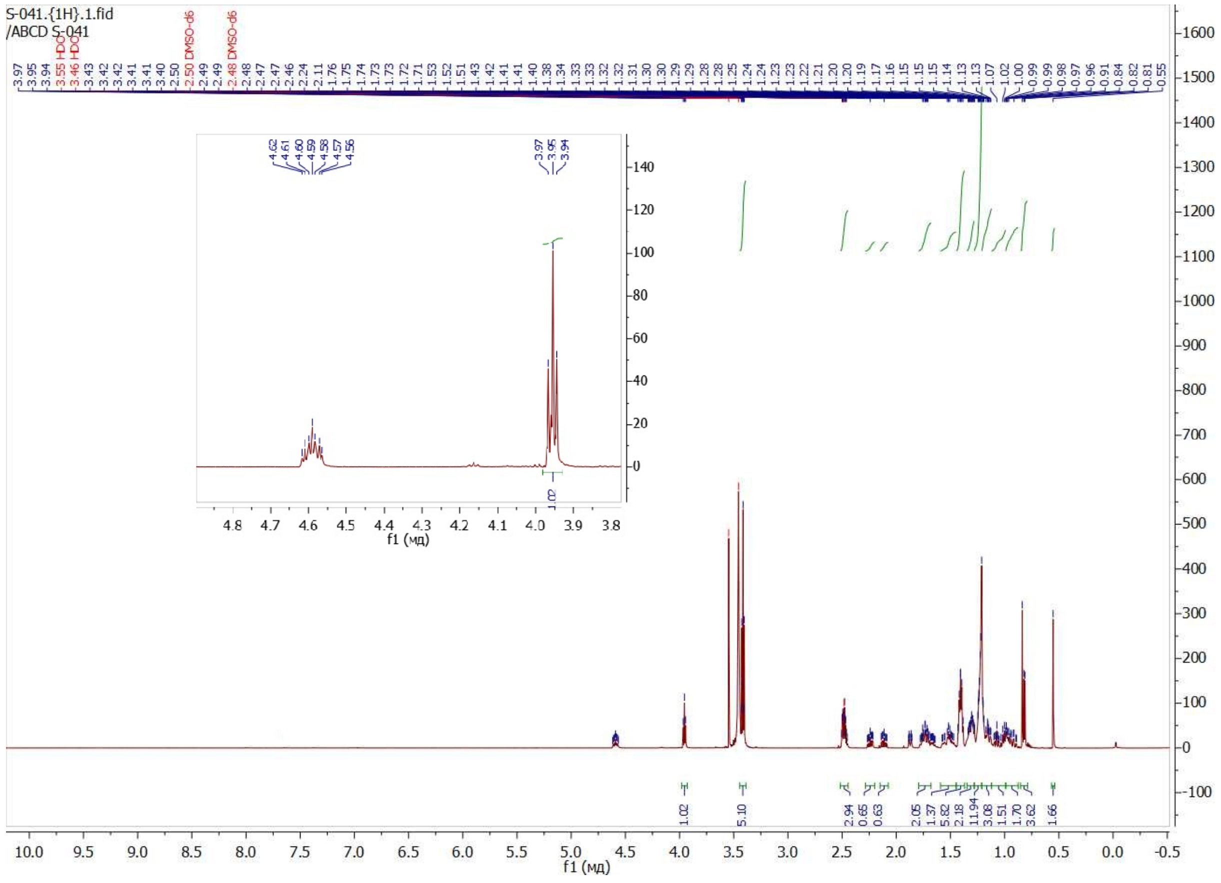

S3. ^{1}H NMR spectrum of compound **3b**.

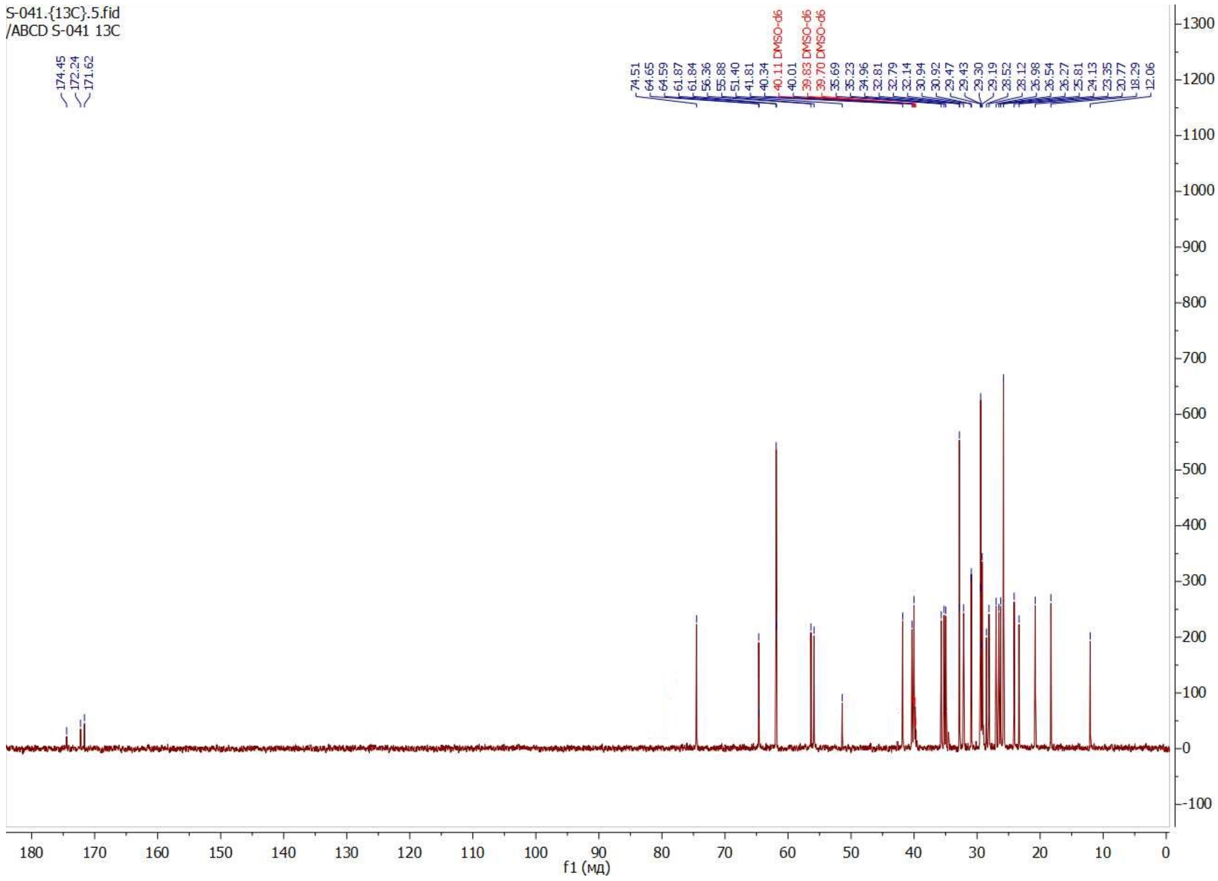

S3. ^{13}C NMR spectrum of compound **3b**.

Display Report

Analysis Info

Analysis Name	D:\Data\Kolotyrkina\2024\Chobanov\1111012.d	Acquisition Date	11.11.2024 13:01:31
Method	tune_low.m	Operator	BDAL@DE
Sample Name	/ABCD S-41	Instrument / Ser#	micrOTOF 10248
Comment	C37H62O7 clb added CH3OH		

Acquisition Parameter

Source Type	ESI	Ion Polarity	Positive	Set Nebulizer	0.4 Bar
Focus	Not active			Set Dry Heater	180 °C
Scan Begin	50 m/z	Set Capillary	4500 V	Set Dry Gas	4.0 l/min
Scan End	3000 m/z	Set End Plate Offset	-500 V	Set Divert Valve	Waste

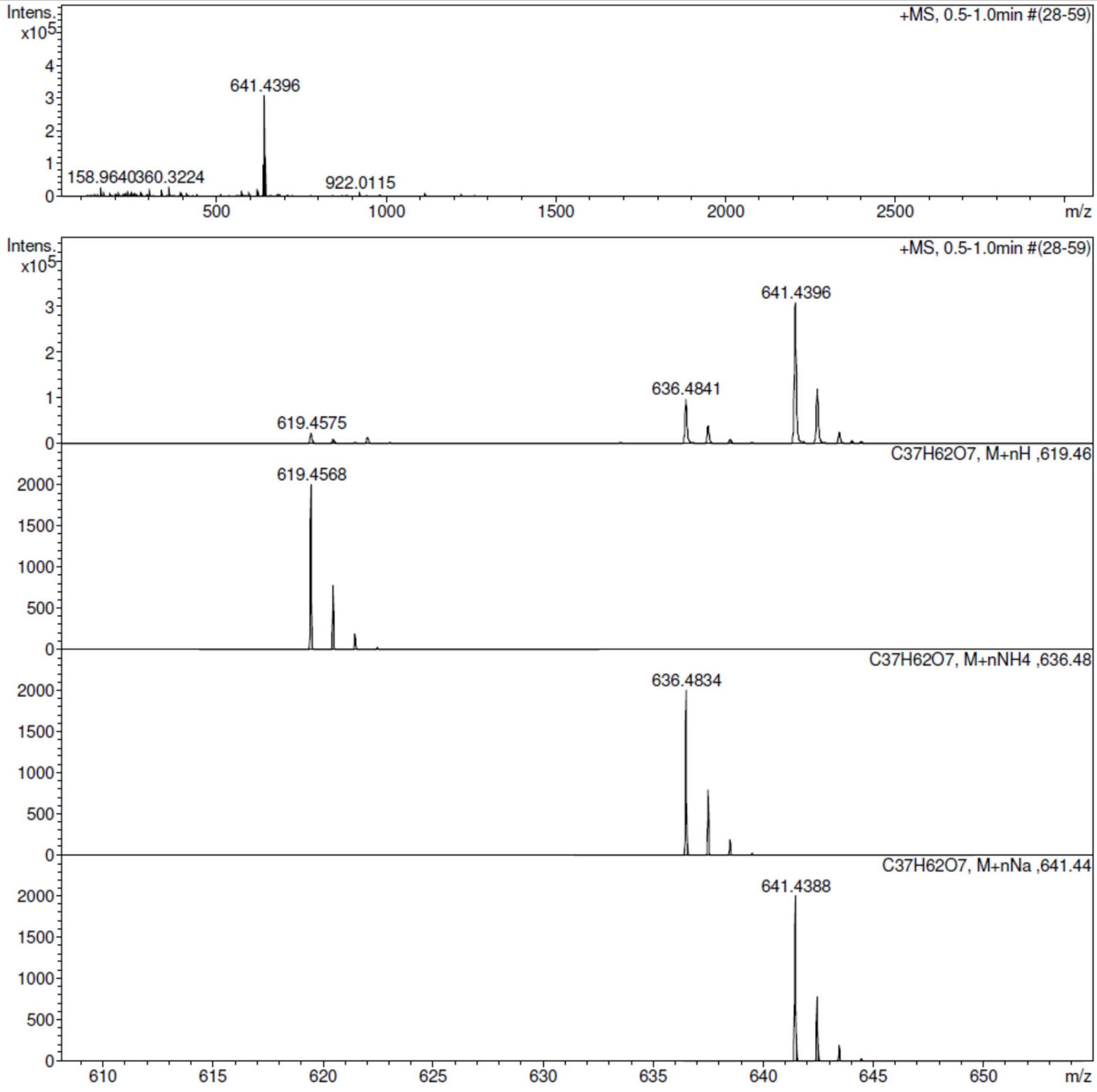

Bruker Compass DataAnalysis 4.0 printed: 11.11.2024 13:04:28 Page 1 of 1

S3. Mass spectrum of compound **3b**.

*General procedure for the synthesis of pyrazole derivatives of lithocholic acid **4a, 4b**.*

To a solution of 6-hydroxyhexyl 3α-(hydroxysuccinyl)oxy-5β-cholan-24-oate **3a** (414 mg, 0.7 mmol) or 8-hydroxyoctyl 3α-(hydroxysuccinyl)oxy-5β-cholan-24-oate **3b** (433 mg, 0.7 mmol) in anhydrous dichloromethane (20 ml), 1-methyl-1*H*-pyrazole-5-carboxylic acid (130 mg, 1.1 mmol) was added, followed by *N*-[3-(methylamino)propyl]-*N*'-ethylcarbodiimide hydrochloride (EDC·HCl) (210 mg, 1.1 mmol) and 4-dimethylaminopyridine (DMAP) (43 mg, 0.35 mmol) under argon. After stirring at room temperature overnight, the mixture was diluted with H_2O (10 ml) and the CH_2Cl_2 layer was separated, dried over $MgSO_4$, and concentrated. The crude product was purified by column chromatography (silica gel) using petroleum ether/EtOAc = 1/2 as the elution solvent to afford the pyrazole derivatives of lithocholic acid **4a**, **4b**.

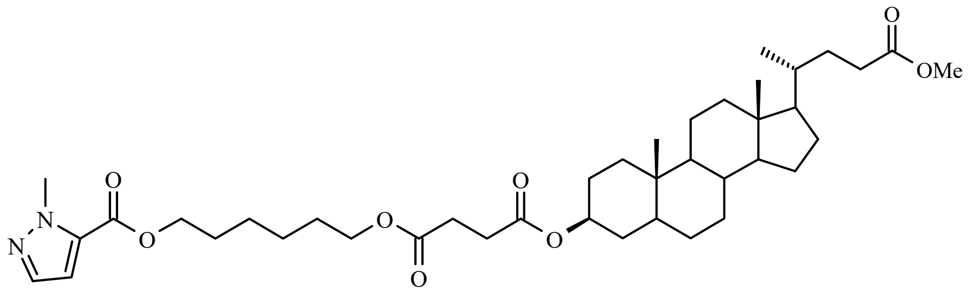

4a

*6-{[(1-Methyl-1H-pyrazol-5-yl)carbonyl]oxy}hexyl(3α)-(succinyl)oxy-5β-cholan-24-oate **(4a)**.*

^{1}H NMR (600 MHz, Chloroform-*d*) δ 4.19 – 4.04 (m, 3H), 3.66 (s, 4H), 2.64 – 2.56 (m, 2H), 2.39 – 2.31 (m, 1H), 2.27 – 2.17 (m, 2H), 1.99 – 1.92 (m, 2H), 1.90 – 1.74 (m, 7H), 1.60 – 1.50 (m, 1H), 1.46 – 1.30 (m, 11H), 1.30 – 1.23 (m, 7H), 1.23 – 1.10 (m, 3H), 1.10 – 1.03 (m, 6H), 1.03 – 0.97 (m, 1H), 0.93 – 0.83 (m, 9H), 0.64 (s, 3H).

^{13}C NMR (151 MHz, $CDCl_3$) δ 174.72, 172.39, 171.78, 137.76, 111.14, 107.09, 74.71, 64.58, 60.60, 56.43, 55.94, 51.44, 42.68, 41.85, 40.37, 40.08, 35.74, 35.33, 34.96, 34.54, 32.15, 31.02, 30.96, 29.50, 29.47, 29.23, 29.18, 28.45, 28.14, 26.96, 26.55, 26.27, 25.54, 24.14, 23.28, 20.79, 18.23, 14.18, 11.99.

MS (ESI): 721.4405 [M+Na]. Calculated for $C_{40}H_{62}N_2O_8Na$: 721.4404.

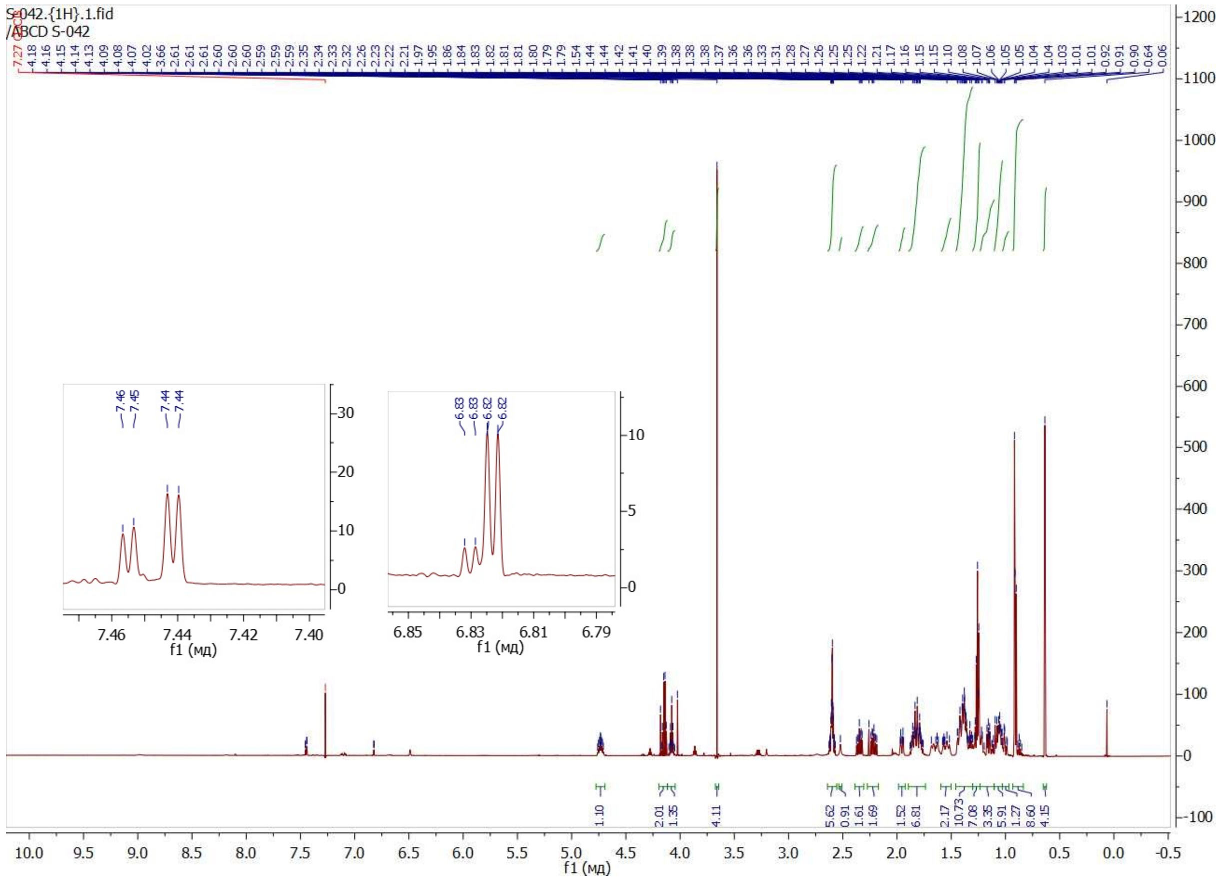

S4. ^{1}H NMR spectrum of compound **4a**.

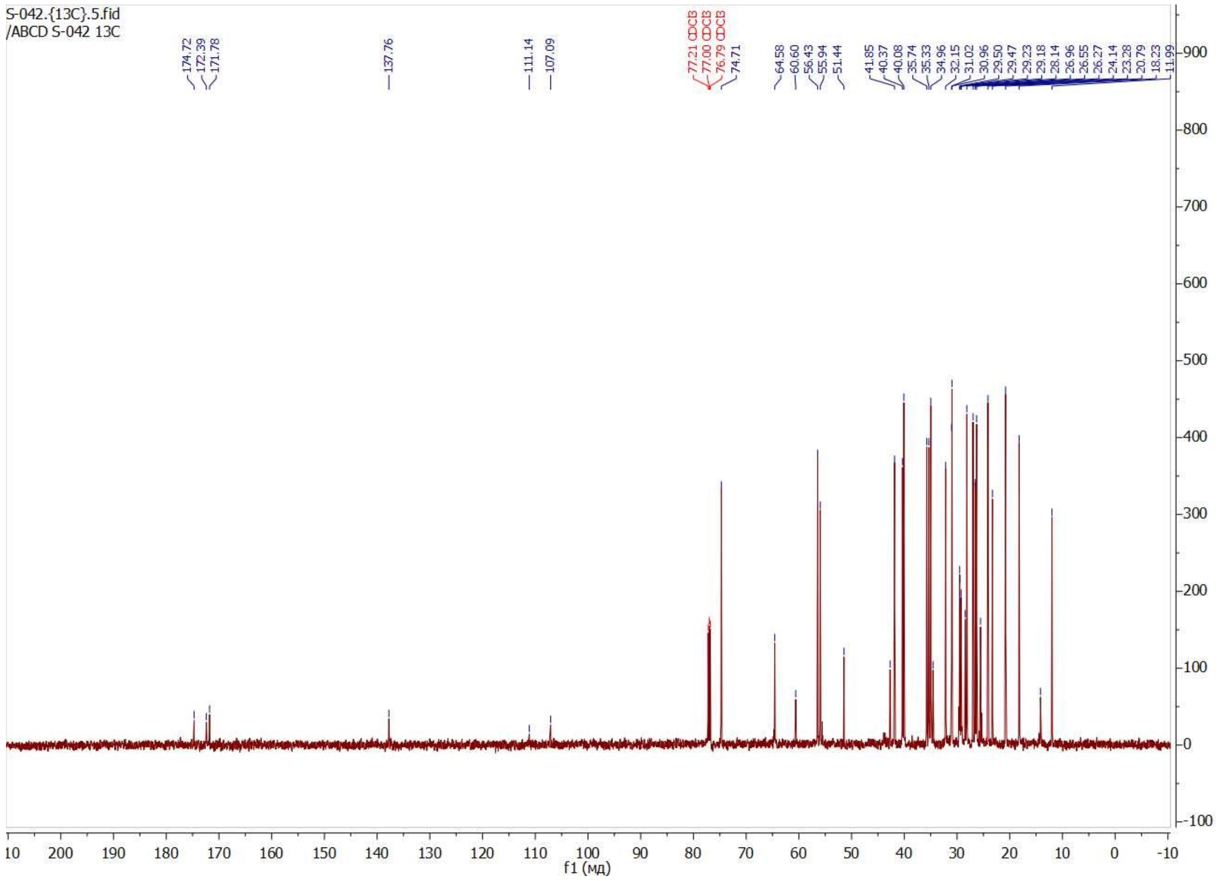

S4. ^{13}C NMR spectrum of compound **4a**.

Display Report

**Analysis Info**		Acquisition Date	12.11.2024 12:57:06
Analysis Name	D:\Data\Kolotyrkina\2024\Chobanov\1112015.d		
Method	tune_low.m	Operator	BDAL@DE
Sample Name	/ABCD S-042	Instrument / Ser#	micrOTOF 10248
Comment	C40H62N2O8 clb added CH3OH		

**Acquisition Parameter**					
Source Type	ESI	Ion Polarity	Positive	Set Nebulizer	0.4 Bar
Focus	Not active			Set Dry Heater	180 °C
Scan Begin	50 m/z	Set Capillary	4500 V	Set Dry Gas	4.0 l/min
Scan End	3000 m/z	Set End Plate Offset	-500 V	Set Divert Valve	Waste

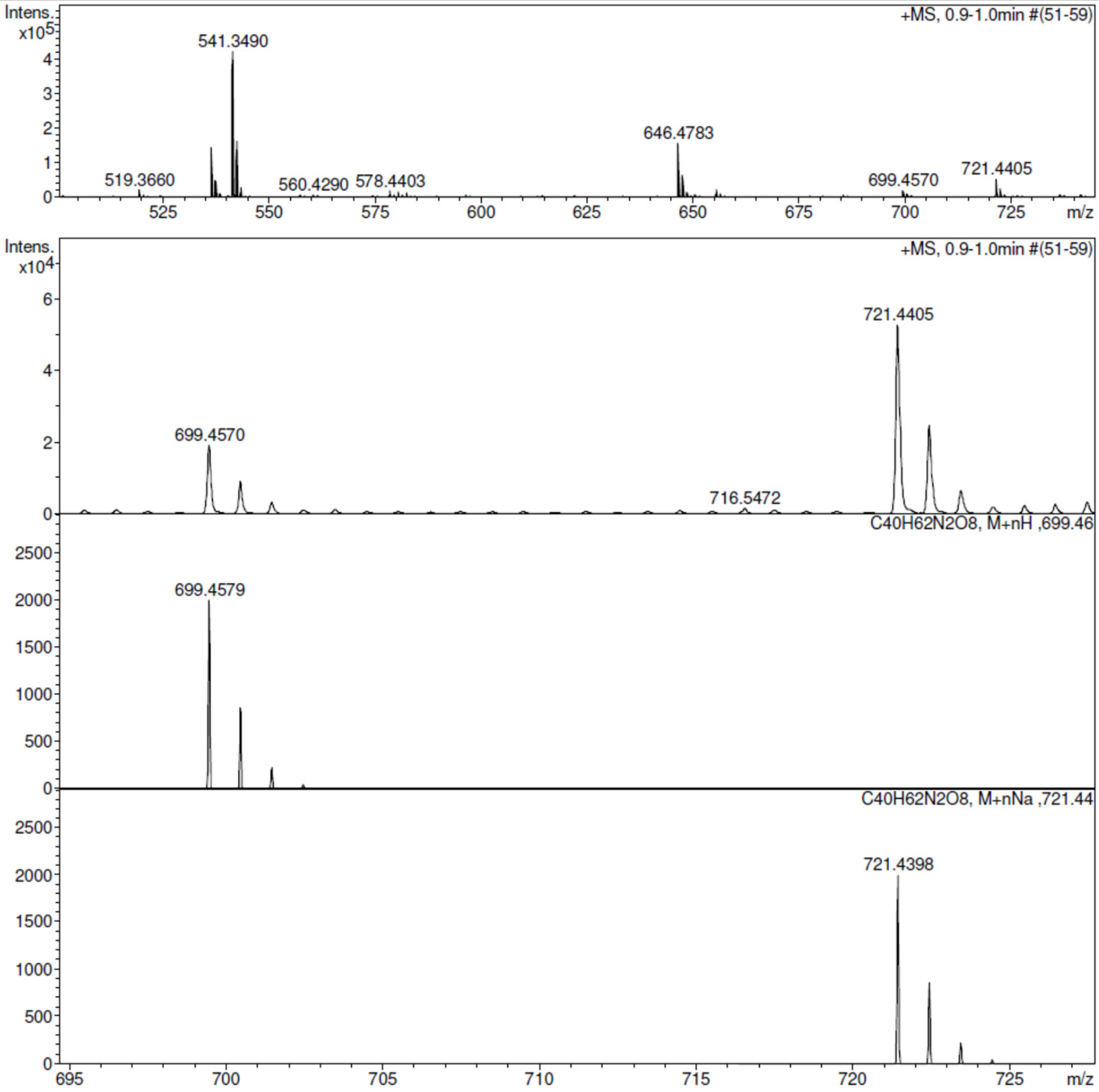

S4. Mass spectrum of compound **4a**.

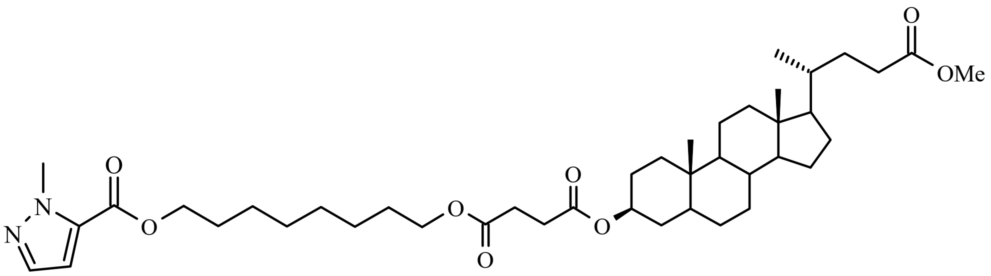

4b

8-{[(1-Methyl-1H-pyrazol-5-yl)carbonyl]oxy}octyl(3α)-(succinyl)oxy-5β-cholan-24-oate ***(4b)***.

^{1}H NMR (300 MHz, Chloroform-*d*) δ 7.37 (d, *J* = 2.1 Hz, 1H), 7.09 – 6.96 (m, 2H), 6.74 (s, 1H), 4.73 – 4.59 (m, 1H), 4.20 (t, *J* = 6.7 Hz, 2H), 4.10 (s, 3H), 4.00 (t, *J* = 6.8 Hz, 2H), 3.61 – 3.50 (m, 4H), 2.53 (s, 3H), 2.32 – 2.21 (m, 1H), 2.18 (s, 3H), 1.99 – 1.78 (m, 2H), 1.76 – 1.59 (m, 2H), 1.59 – 1.43 (m, 4H), 1.39 – 1.22 (m, 18H), 1.16 (q, *J* = 10.9, 8.7 Hz, 5H), 1.08 – 0.95 (m, 4H), 0.95 – 0.79 (m, 6H), 0.57 (s, 3H).

^{13}C NMR (75 MHz, $CDCl_3$) δ 174.73, 172.43, 171.81, 159.96, 137.73, 129.59, 125.80, 111.18, 74.78, 65.09, 65.05, 65.02, 64.83, 64.77, 62.91, 56.54, 56.07, 51.46, 42.78, 41.96, 40.49, 40.18, 39.53, 35.84, 35.40, 35.06, 34.62, 32.79, 32.76, 32.26, 31.10, 31.06, 29.71, 29.62, 29.60, 29.33, 29.31, 29.21, 29.14, 29.12, 28.61, 28.21, 27.06, 26.65, 26.37, 25.91, 25.84, 25.71, 24.21, 23.35, 20.88, 19.72, 18.31, 12.07.

<u>MS (ESI)</u>: 749.4715 [M+Na]. Calculated for $C_{42}H_{66}N_2O_8Na$: 749.4716.

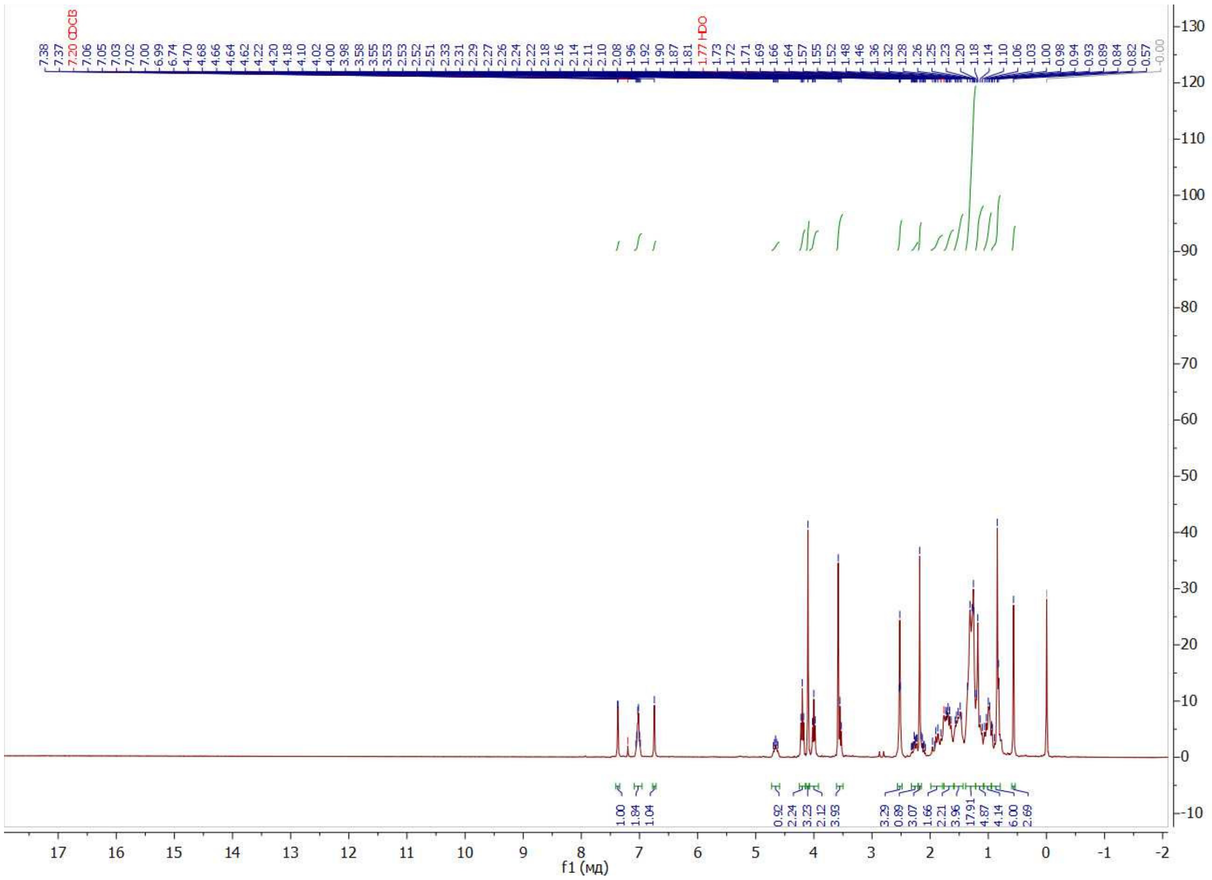

S5. ^{1}H NMR spectrum of compound **4b**.

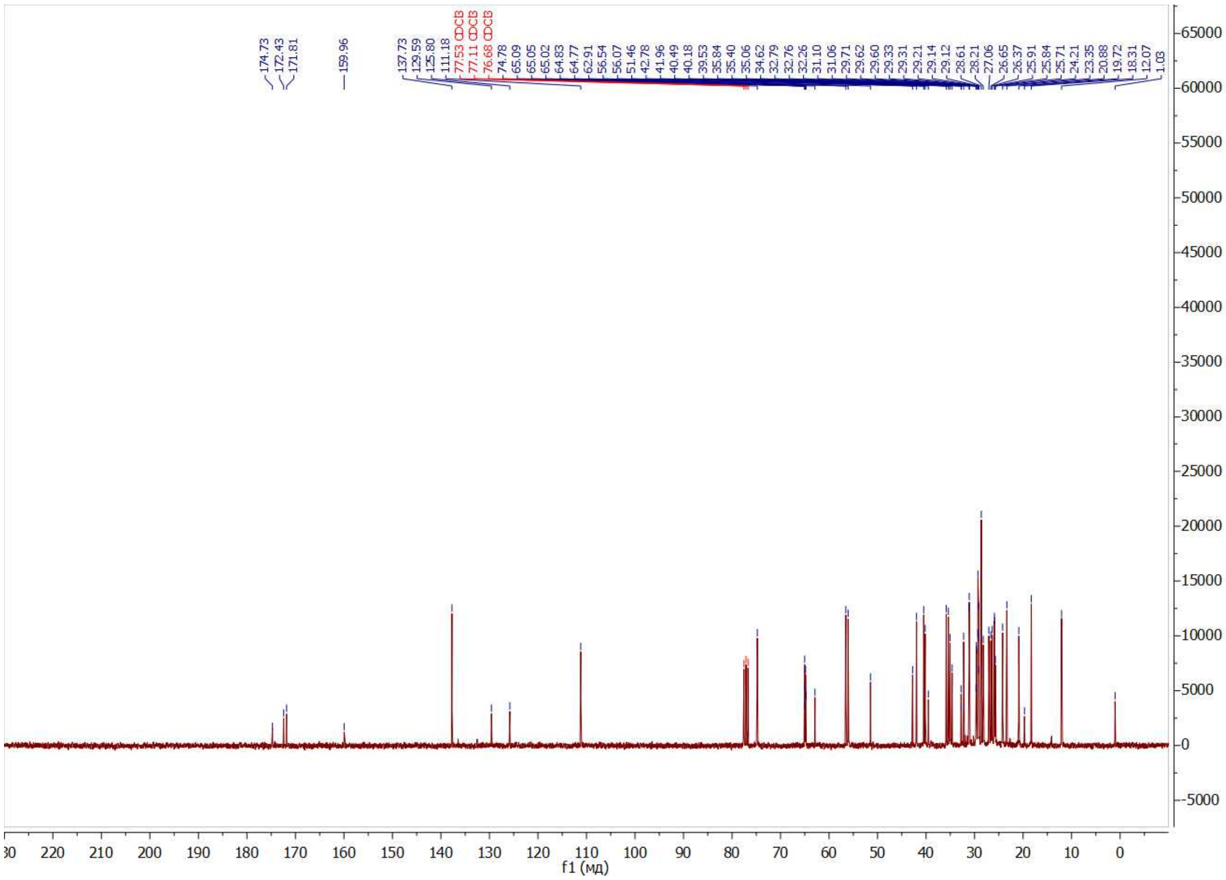

S5. ^{13}C NMR spectrum of compound **4b**.

Display Report

Analysis Info

Analysis Name	D:\Data\Kolotyrkina\2024\Chobanov\1111014.d	Acquisition Date	11.11.2024 13:17:05
Method	tune_low.m	Operator	BDAL@DE
Sample Name	/ABCD S-056-2	Instrument / Ser#	micrOTOF 10248
Comment	C42H66N2O8 clb added CH3OH		

Acquisition Parameter

Source Type	ESI	Ion Polarity	Positive	Set Nebulizer	0.4 Bar
Focus	Not active			Set Dry Heater	180 °C
Scan Begin	50 m/z	Set Capillary	4500 V	Set Dry Gas	4.0 l/min
Scan End	3000 m/z	Set End Plate Offset	-500 V	Set Divert Valve	Waste

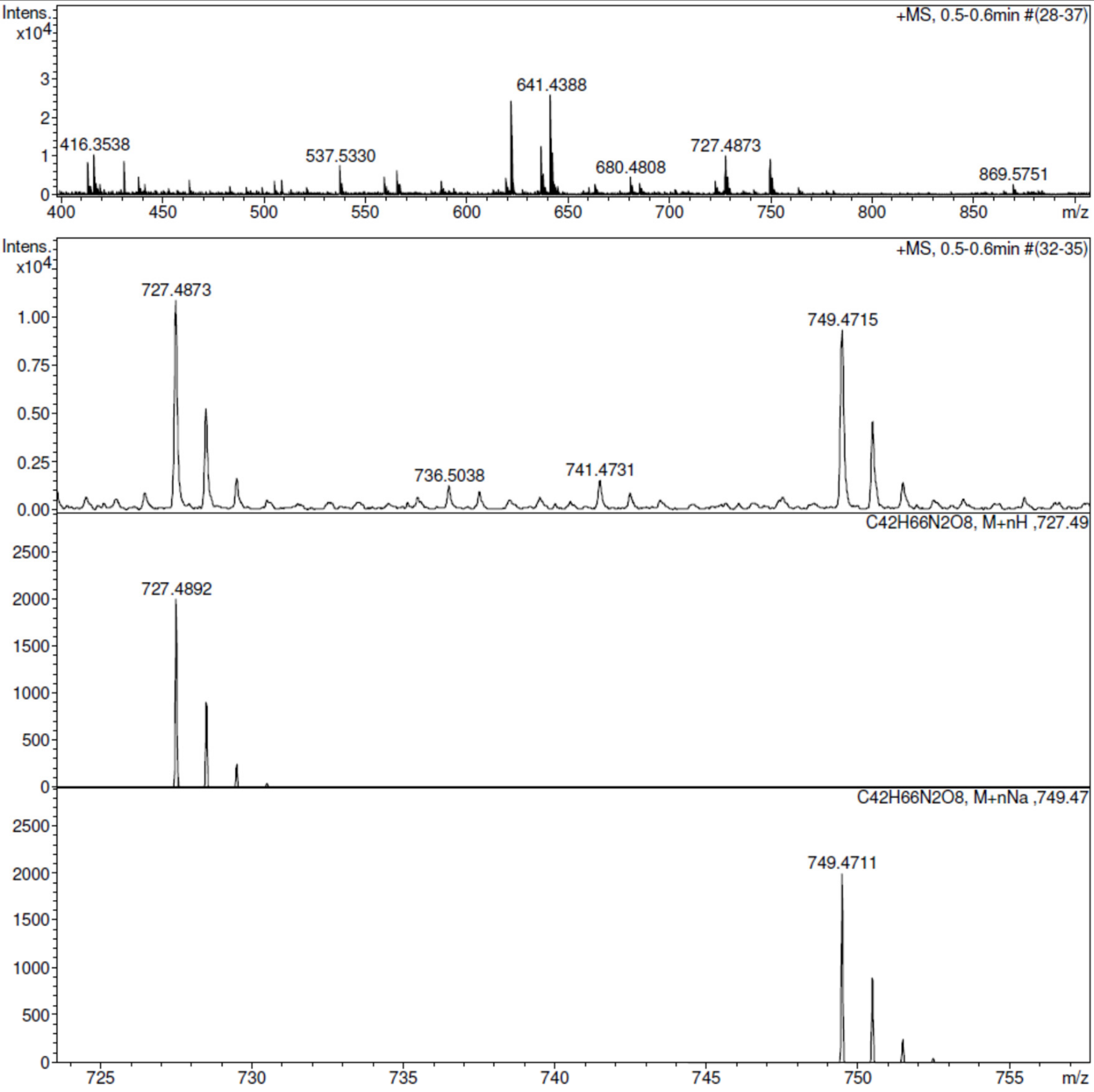

S5. Mass spectrum of compound **4b**.

*General procedure for the synthesis of ionic compounds based on pyrazole derivatives of lithocholic acid **5a, 5b**.*

To a solution of pyrazole derivatives of lithocholic acid **4a** (421 mg, 0.5 mmol) or **4b** (435 mg, 0.5 mmol), respectively) in anhydrous dichloromethane (10 ml), methyl iodide (CH_3I) (71 mg, 0.5 mmol) was added under argon. The mixture was stirred at room temperature overnight. After stirring, the solvent was evaporated. The crude product was purified by column chromatography (silica gel) using petroleum ether/EtOAc = 1/1 as the elution solvent to afford ionic compounds of pyrazole derivatives of lithocholic acid **5a, 5b**.

*6-{[(1,2-dimethyl-1H-pyrazolyliodide)carbonyl]oxy}hexyl(3α)-(*succinyl)oxy-5β-cholan-24-oate (***5a)***.*

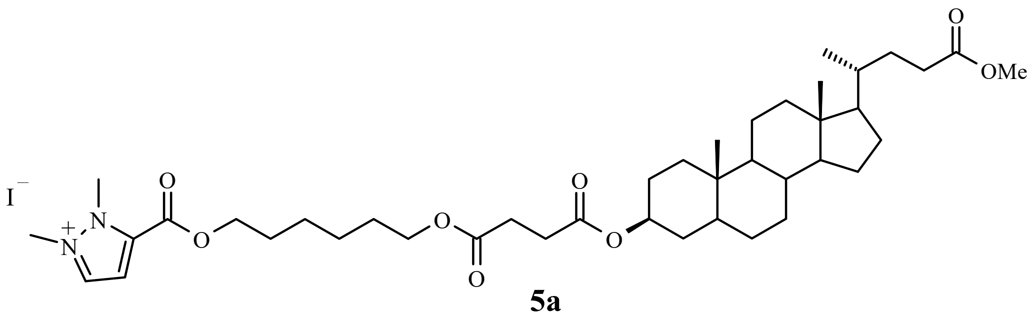

5a

^{1}H NMR (300 MHz, DMSO-*d*6) δ 4.74 – 4.46 (m, 3H), 4.12 – 3.95 (m, 3H), 3.58 (s, 4H), 3.11 (s, 1H), 2.37 – 2.08 (m, 2H), 2.02 – 0.92 (m, 31H), 0.93 – 0.83 (m, 7H), 0.62 (s, 4H).

^{13}C NMR (75 MHz, DMSO-*d*6) δ 173.38, 171.40, 170.84, 136.63, 73.61, 63.68, 59.71, 55.78, 55.32, 50.81, 42.11, 41.15, 40.34, 40.06, 39.78, 39.50, 39.22, 38.94, 38.67, 35.14, 34.61, 34.38, 33.99, 31.64, 30.40, 30.23, 28.83, 28.62, 28.56, 27.90, 27.53, 26.45, 26.02, 25.74, 24.92, 23.60, 22.83, 20.23, 17.85, 13.83, 11.60.

MS (ESI): $C_{41}H_{65}N_2O_8$: 713.4734 [M]. Calculated for $C_{41}H_{65}N_2O_8$: 713.4740.

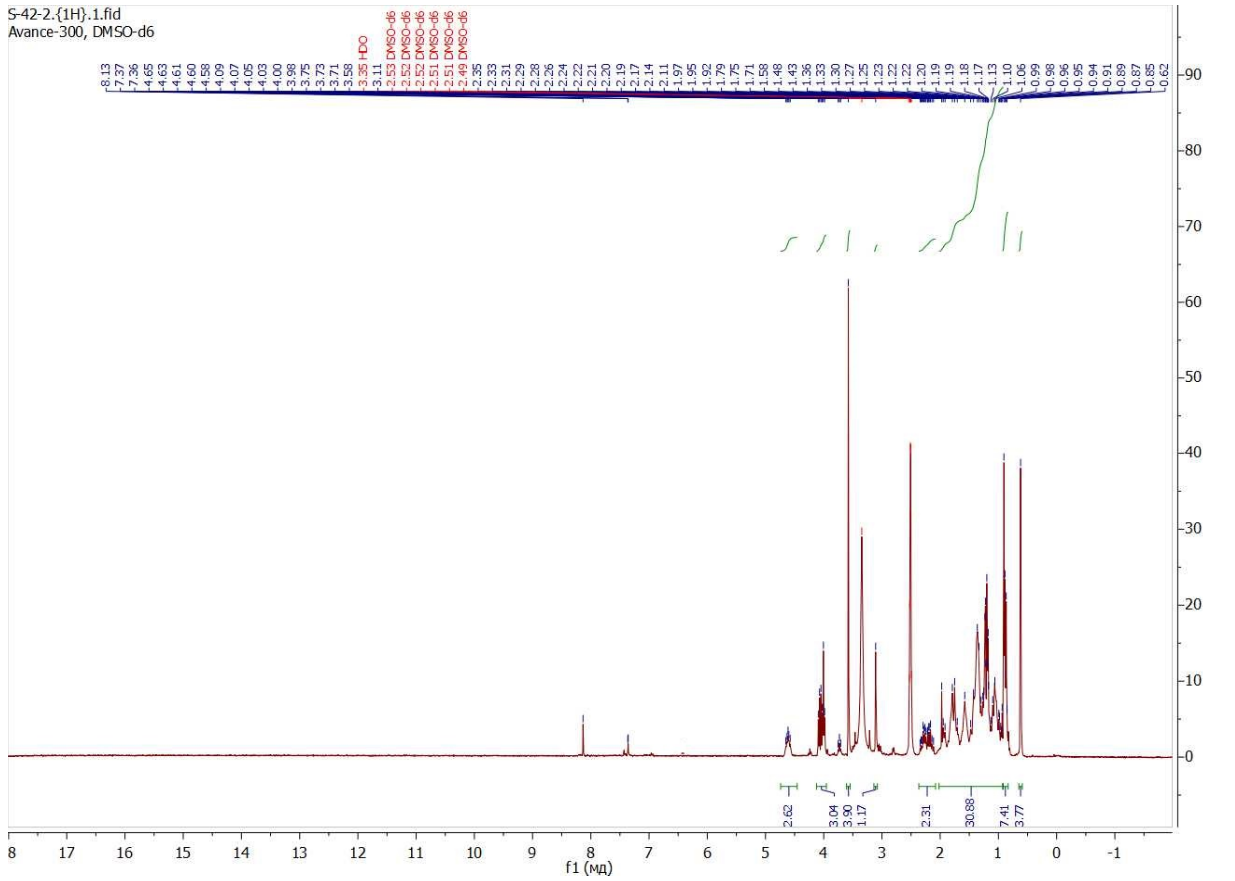

S6. ^{1}H NMR spectrum of compound **5a**.

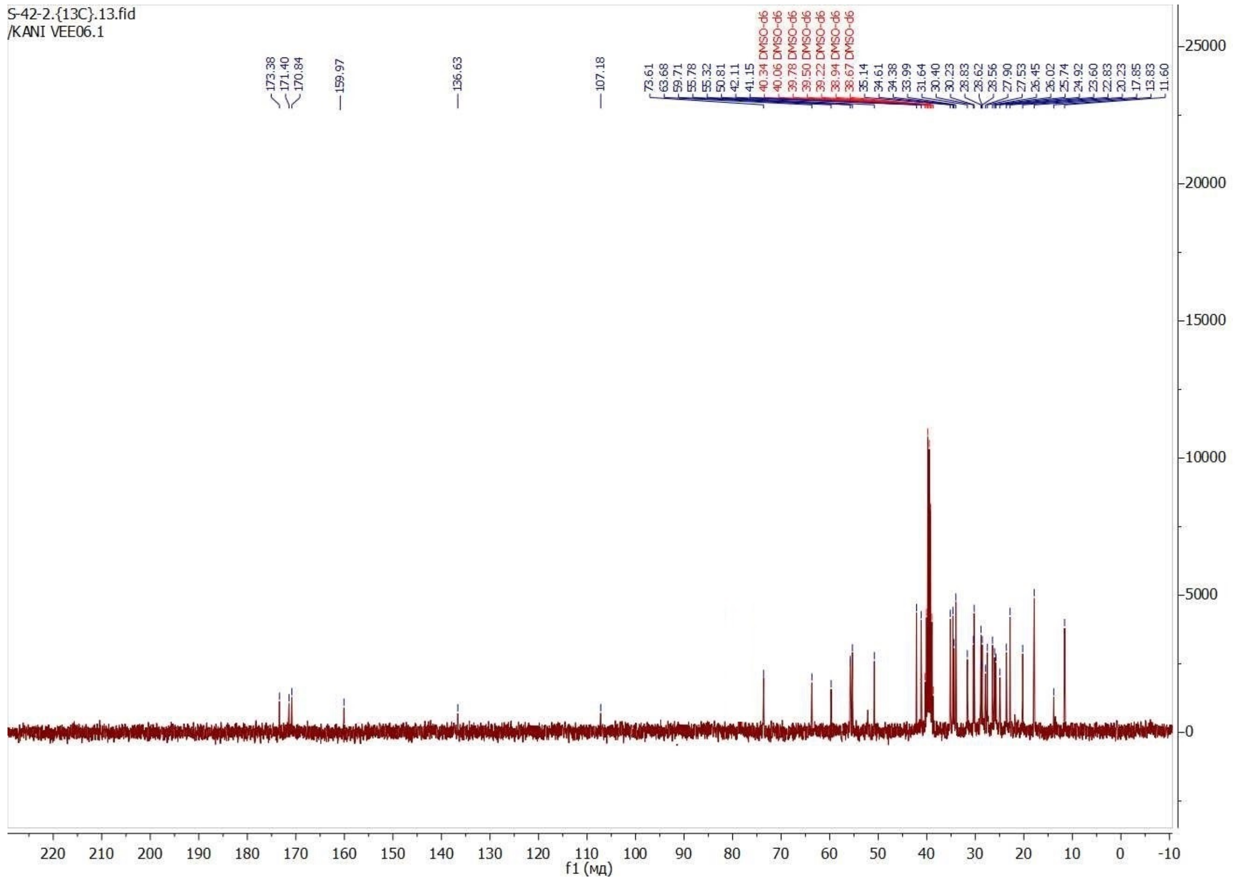

S6. ^{13}C NMR spectrum of compound **5a**.

Display Report

Analysis Info

Analysis Name	D:\Data\Kolotyrkina\2024\Chobanov\1107003.d	Acquisition Date	07.11.2024 12:20:03
Method	tune_low.m	Operator	BDAL@DE
Sample Name	/ABCD S-42-2	Instrument / Ser#	micrOTOF 10248
Comment	C41H65N2O8 clb added CH3OH		

Acquisition Parameter

Source Type	ESI	Ion Polarity	Positive	Set Nebulizer	0.4 Bar
Focus	Not active			Set Dry Heater	180 °C
Scan Begin	50 m/z	Set Capillary	4500 V	Set Dry Gas	4.0 l/min
Scan End	3000 m/z	Set End Plate Offset	-500 V	Set Divert Valve	Waste

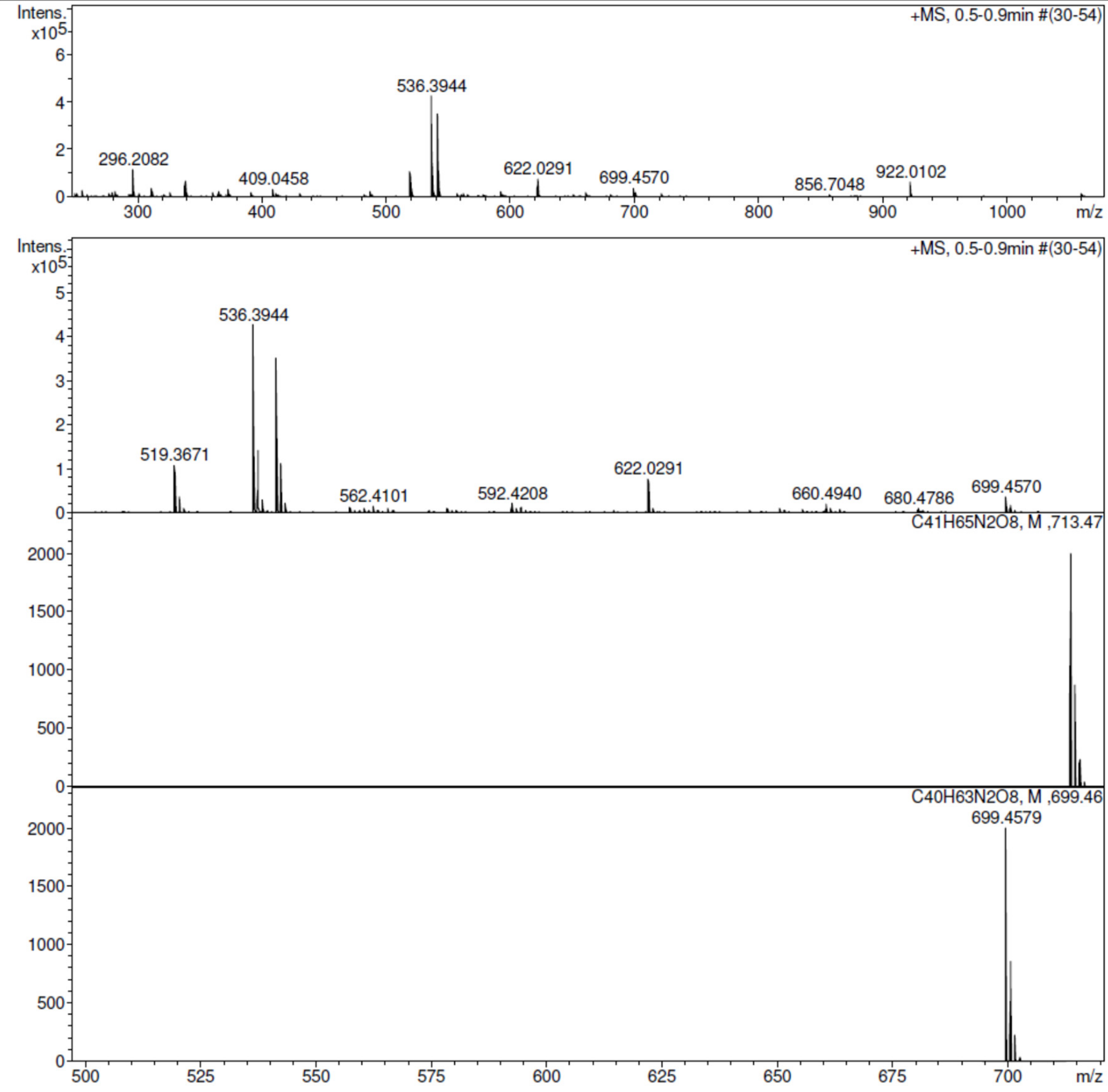

Bruker Compass DataAnalysis 4.0 printed: 07.11.2024 12:24:39 Page 1 of 1

S6. Mass spectrum of compound **5a**.

6-{[(1,2-dimethyl-1H-pyrazolyliodide)carbonyl]oxy}octyl(3α)-(succinyl)oxy-5β-cholan-24-oate ***(5b)***.

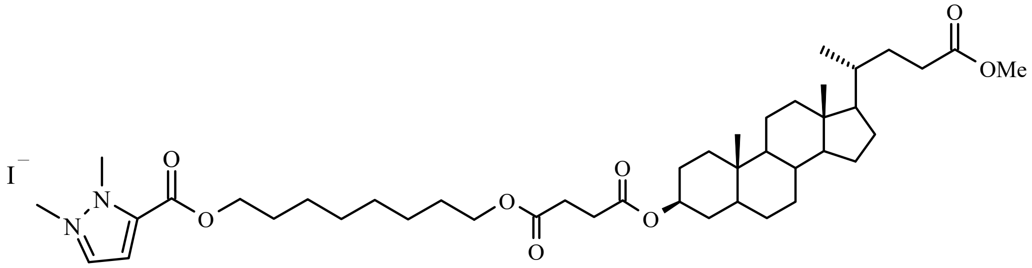

5b

^{1}H NMR (300 MHz, Chloroform-*d*) δ 4.81 – 4.67 (m, 1H), 4.28 (t, *J* = 6.7 Hz, 4H), 4.19 (s, 6H), 4.08 (t, *J* = 6.8 Hz, 3H), 3.70 – 3.59 (m, 6H), 2.64 – 2.58 (m, 4H), 2.41 – 2.16 (m, 4H), 2.01 – 1.81 (m, 4H), 1.81 – 1.68 (m, 6H), 1.68 – 1.52 (m, 6H), 1.39 – 1.30 (m, 13H), 1.29 – 1.19 (m, 4H), 1.19 – 0.98 (m, 6H), 0.91 (d, *J* = 6.4 Hz, 6H), 0.65 (s, 3H).

^{13}C NMR (75 MHz, $CDCl_3$) δ 174.81, 172.49, 171.87, 159.95, 137.69, 132.55, 111.21, 74.81, 65.16, 65.09, 64.86, 62.95, 56.53, 56.05, 51.52, 42.78, 41.95, 40.47, 40.17, 39.56, 35.83, 35.41, 35.06, 34.63, 32.76, 32.25, 31.11, 31.05, 29.73, 29.62, 29.32, 29.21, 29.14, 28.61, 28.23, 27.05, 26.65, 26.37, 25.92, 25.85, 25.71, 24.22, 23.36, 20.88, 18.31, 12.08.

MS (ESI): $C_{43}H_{69}N_2O_8$:741.5048 [M]. Calculated for $C_{43}H_{69}N_2O_8$: 741.5053.

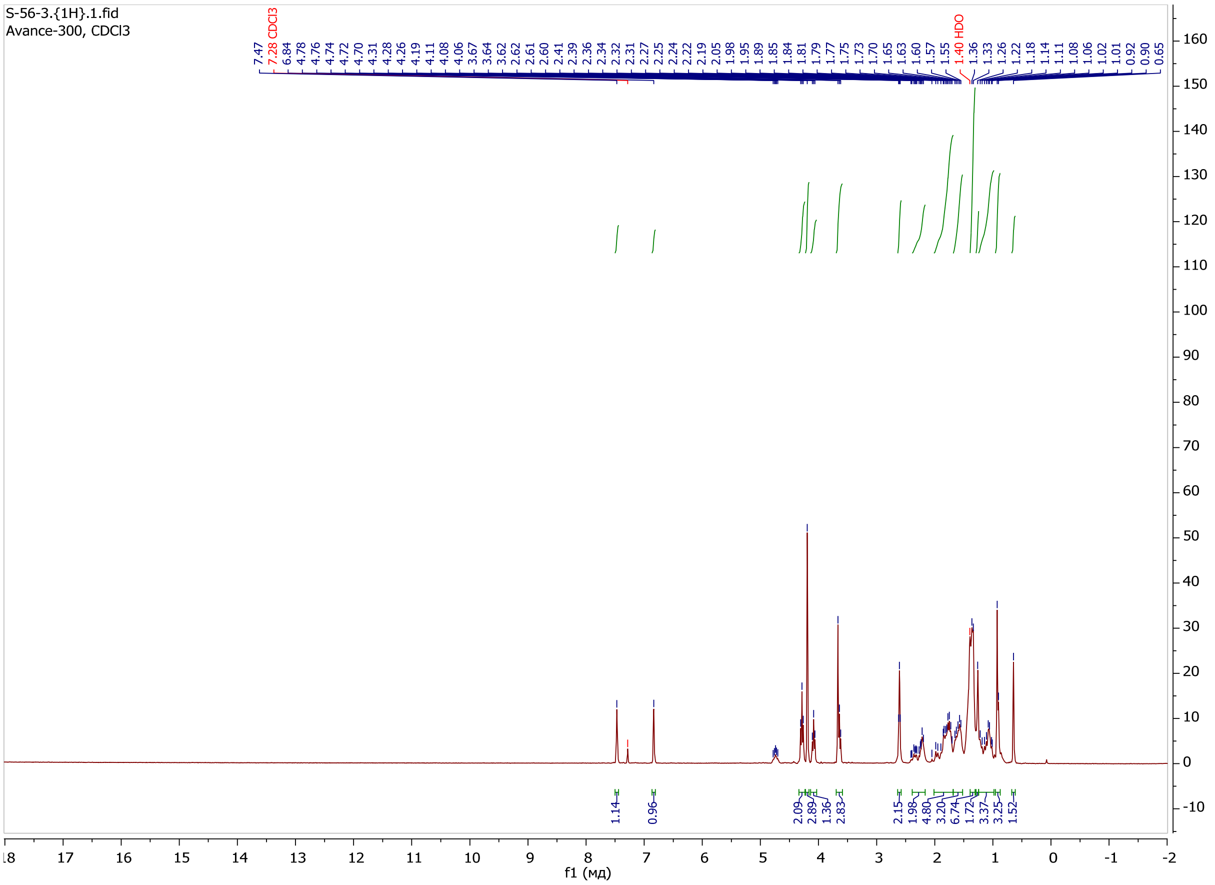

S7. ^{1}H NMR spectrum of compound **5b**.

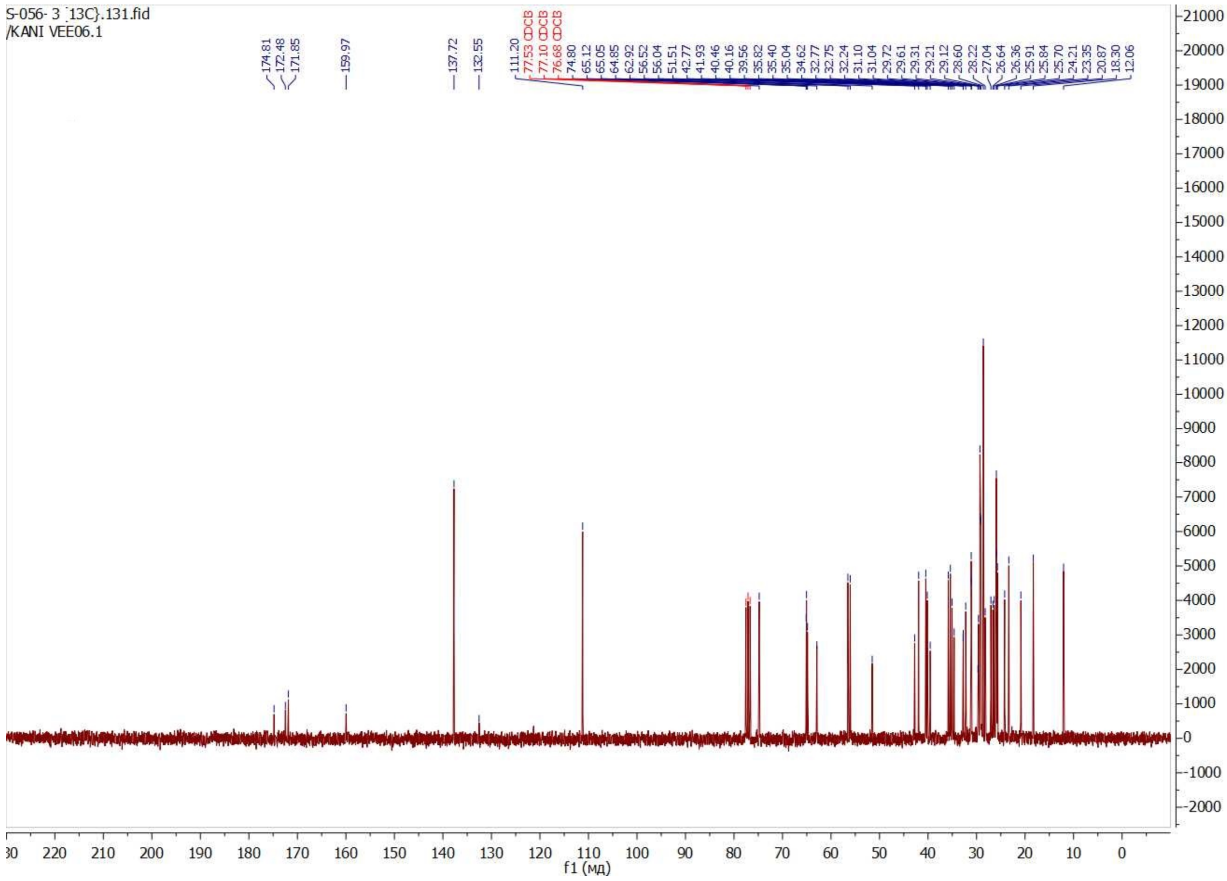

S7. ^{13}C NMR spectrum of compound **5b**.

Display Report

Analysis Info

Analysis Name	D:\Data\Kolotyrkina\2024\Chobanov\1111013.d	Acquisition Date	11.11.2024 13:06:16
Method	tune_low.m	Operator	BDAL@DE
Sample Name	/ABCD S-56-3	Instrument / Ser#	micrOTOF 10248
Comment	C43H69N2O8 clb added CH3OH		

Acquisition Parameter

Source Type	ESI	Ion Polarity	Positive	Set Nebulizer	0.4 Bar
Focus	Not active			Set Dry Heater	180 °C
Scan Begin	50 m/z	Set Capillary	4500 V	Set Dry Gas	4.0 l/min
Scan End	3000 m/z	Set End Plate Offset	-500 V	Set Divert Valve	Waste

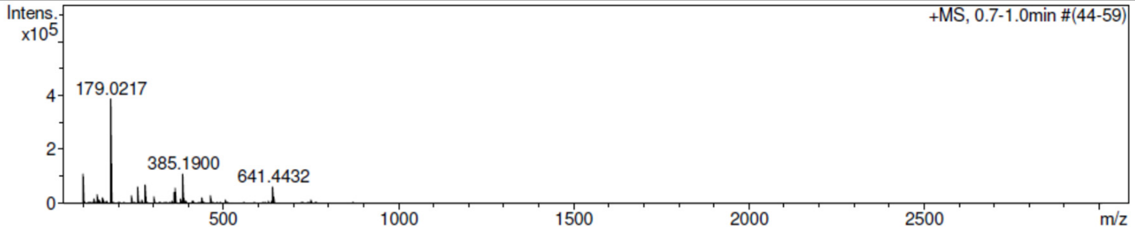

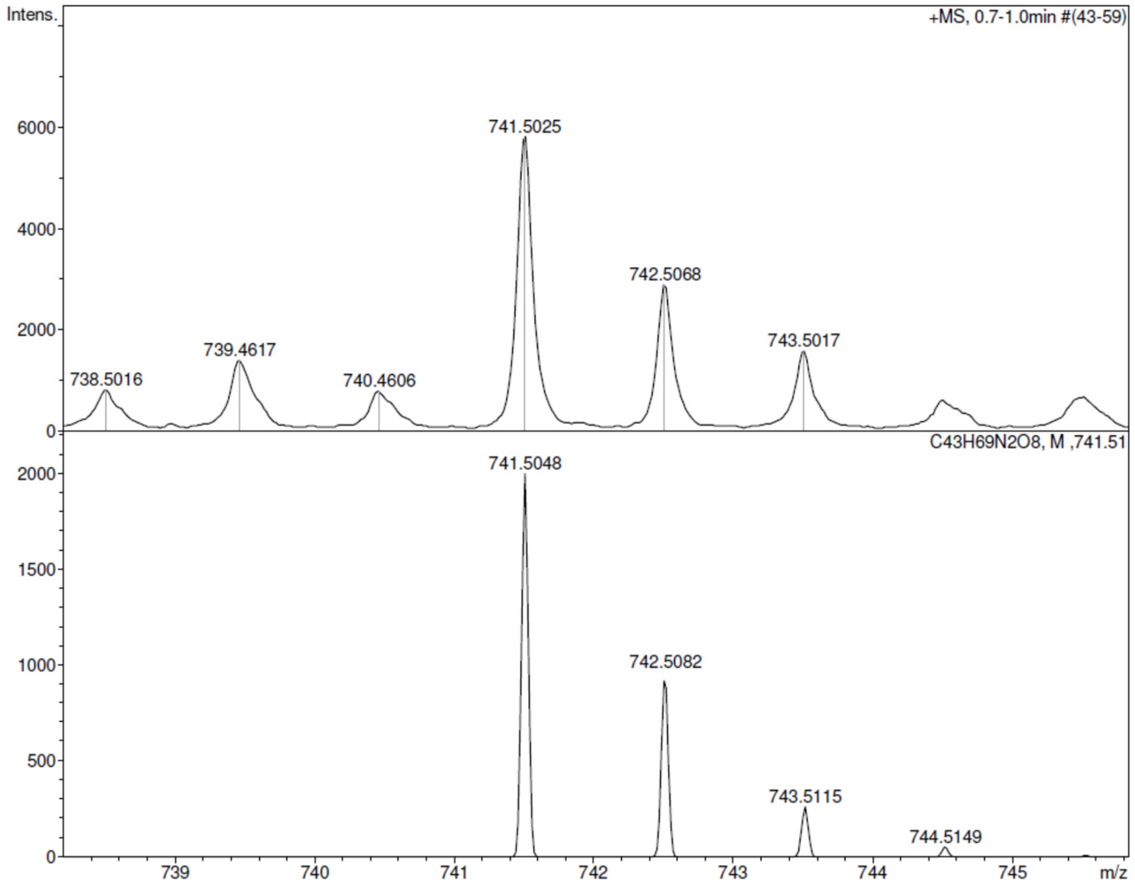

Bruker Compass DataAnalysis 4.0 printed: 11.11.2024 13:14:41 Page 1 of 1

S7. Mass spectrum of compound **5b**.

Table S1. Cytotoxic activity dataset of hybrid molecules and ionic compounds measured in this study (mM).

	Jurkat	K562	A549	HEK293
**Str**	44±3	49±4	51±4	54±5
**CPT**	588±49	594±54	599±41	613±57
**2 (S-030-2)**	417±38	456±41	482±46	488±32
**3a (S-040)**	476±34	480±45	483±37	491±43
**4a (S-042)**	214±18	224±24	290±21	301±29
**5a (S-042-2)**	178±15	158±12	188±16	196±11
**3b (S-041)**	661±57	670±49	683±52	701±64
**4b (S-056-2)**	210±18	213±14	215±17	240±21
**5b (S-056-3)**	233±21	254±19	256±23	260±24

Table S2. The dataset comprises measurements of apoptosis induction and cell cycle phases of hybrid molecules and lithocholic acid-based ionic compounds.

Compound	Molar mass (g/mol)	Apoptosis (%)				Mitostress (%)				Cell cycle (%)				Mitopotential (%)			Cytochtome C loss (%)	
		live	early	last	necrosis	Live	Stress	Apoptosis	Apotosis+stress	G1	S	G2\M	preG0	Live	Blok mito	Dead	Live	Mitdead
**Untreated**	-	97.1	0	0	2.87	95.51	0.01	0.43	0.05	75.47	17.44	4.85	2.18	98.22	1.24	0.36	97.64	2.34
**Str**	466.541	88.7	0.26	3.86	7.14	n/a	n/a	n/a	n/f	n/a	n/a	n/a	n/f	n/a	n/a	n/a	n/a	n/a
**CCCP**	204.62	n/a	n/a	n/a	n/f	n/a	n/a	n/a	n/f	n/a	n/a	n/a	n/f	53.17	12.00	22.34	34.15	60.24
**CPT**	348.358	28.0	1.59	11.3	59.1	n/a	n/a	n/a	n/f	61.18	18.29	2.95	16.59	n/a	n/a	n/a	n/a	n/a
**2**	490.68	22.0	1.85	34.9	41.3	53.24	0.18	41.01	5.57	66.18	16.53	2.98	13.60	1.17	46.76	52.53	92.69	6.70
**3a**	576.82	86.0	0.69	4.30	9.06	43.52	0.05	54.17	2.25	81.33	12.89	2.75	2.78	75.06	10.12	13.05	88.90	10.00
**4a**	698.94	88.1	0.95	8.42	1.94	88.55	2.26	3.27	5.93	70.76	11.19	6.44	11.28	65.56	11.59	12.40	83.18	15.66
**5a**	841.89	92.5	0.03	0.34	7.15	31.78	0.53	43.15	24.54	83.17	11.17	3.62	1.86	31.30	50.74	17.63	77.81	21.06
**3b**	604.87	86.2	1.14	8.96	3.68	39.95	2.44	57.56	2.44	85.80	9.64	2.05	2.35	63.22	5.27	17.03	91.22	8.25
**4b**	712.97	85.9	0.15	1.6	12.3	47.31	0.97	41.82	9.91	83.71	7.10	5.57	3.37	77.92	12.57	7.13	87.26	11.66
**5b**	855.92	78.7	11.9	6.9	2.5	36.69	0.12	53.18	10.02	68.02	11.39	6.53	13.25	5.22	46.52	45.95	76.84	22.05

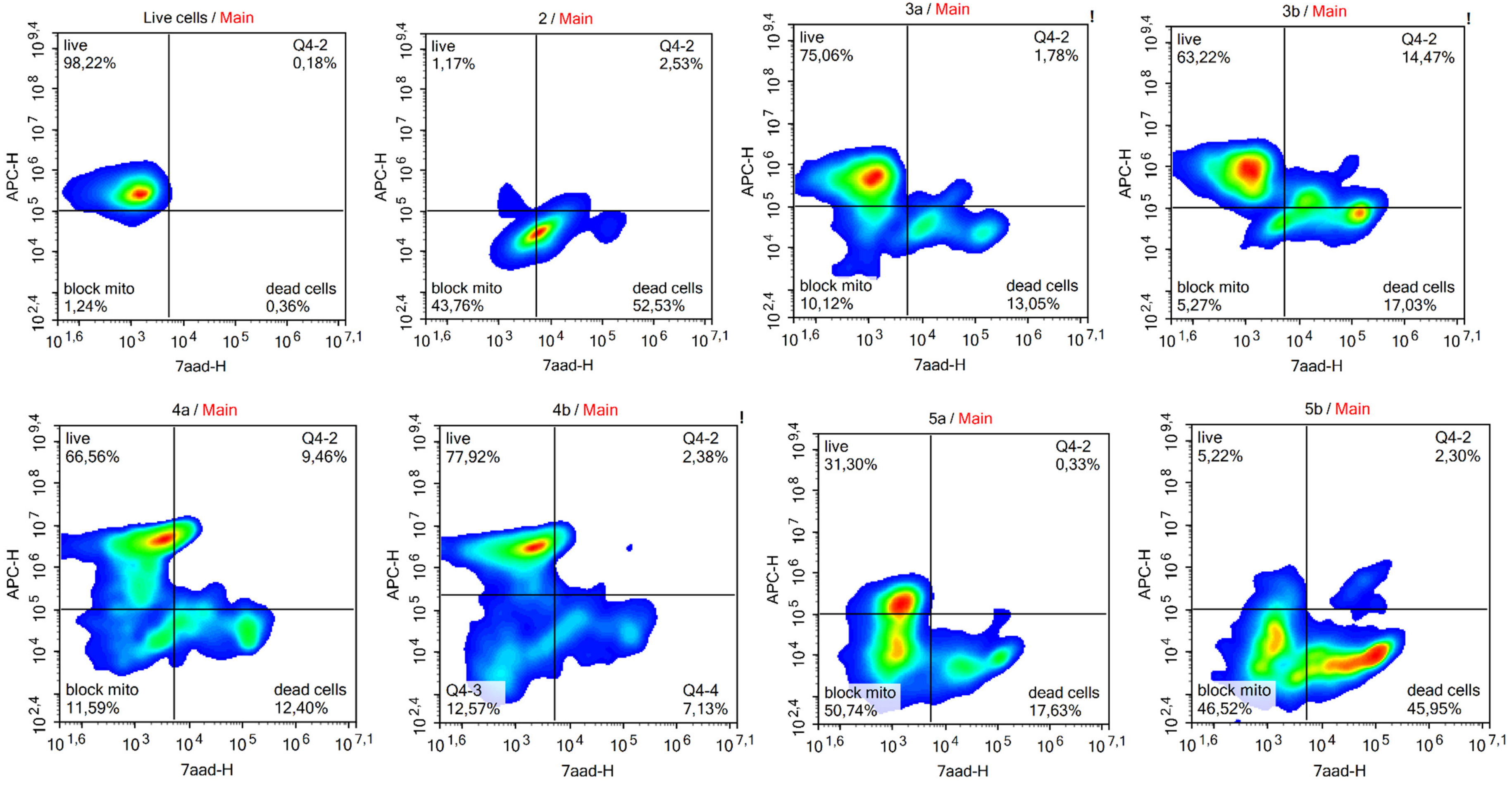
Live cells / Main
live 98,22%
Q4-2 0,18%
block mito 1,24%
dead cells 0,36%
2 / Main
live 1,17%
Q4-2 2,53%
block mito 43,76%
dead cells 52,53%
3a / Main
live 75,06%
Q4-2 1,78%
block mito 10,12%
dead cells 13,05%
3b / Main
live 63,22%
Q4-2 14,47%
block mito 5,27%
dead cells 17,03%
4a / Main
live 66,56%
Q4-2 9,46%
block mito 11,59%
dead cells 12,40%
4b / Main
live 77,92%
Q4-2 2,38%
Q4-3 12,57%
Q4-4 7,13%
5a / Main
live 31,30%
Q4-2 0,33%
block mito 50,74%
dead cells 17,63%
5b / Main
live 5,22%
Q4-2 2,30%
block mito 46,52%
dead cells 45,95%
APC-H
7aad-H

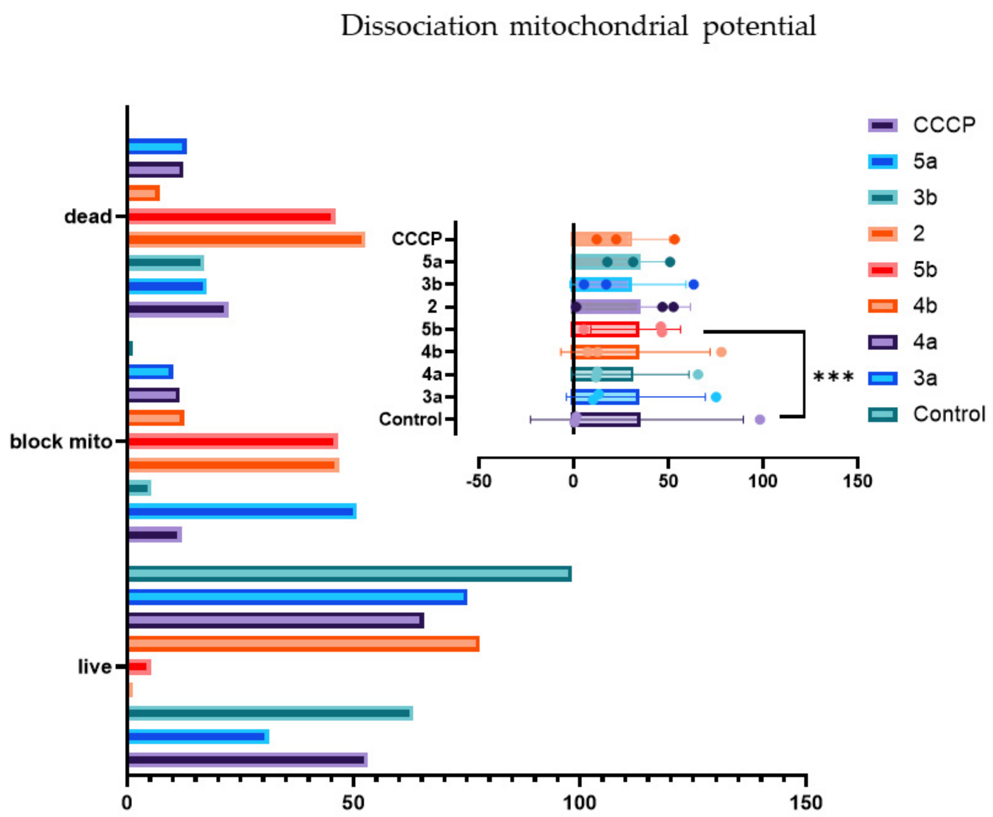

Figure S1. (4) The objective of this study is to simultaneously determine the levels of cell death markers and mitochondrial membrane potential disruption in Jurkat cells exposed to a series of synthesized hybrid molecules and lithocholic acid-based ionic compounds. (A) The number of cells with altered mitochondrial potential (negative for JC-1 and 7-AAD) and necrotic cells (negative for JC-1 and positive for 7-AAD) were observed over a 12-hour period. The concentration of the tested compounds was taken at CC_{50} (the cytotoxicity table of co-compounds is presented in SI). (B) A histogram of mitochondrial potential changes in cells in the Jurkat culture upon exposure to hybrid molecules and lithocholic acid-based ionic compounds is presented.

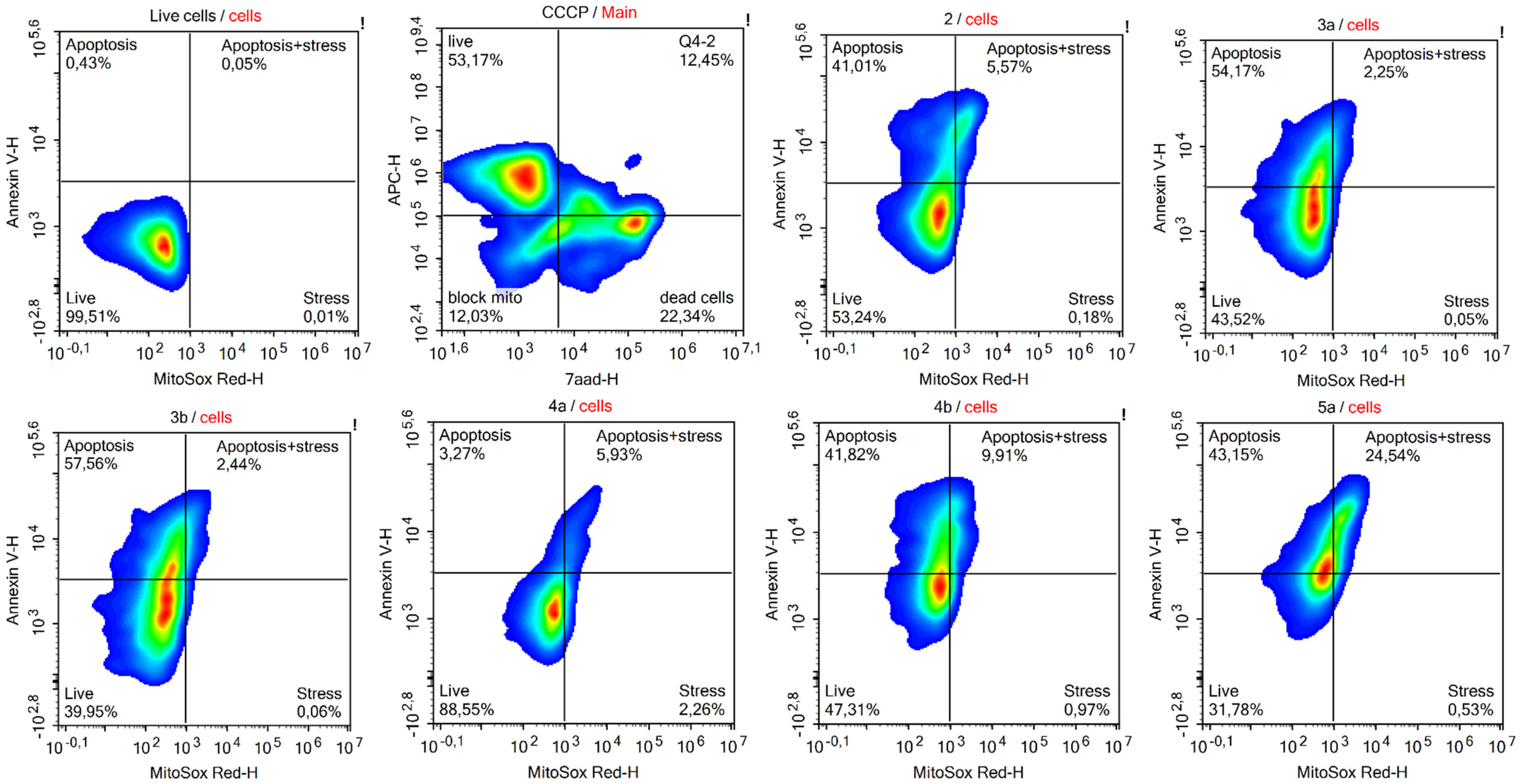

Live cells / cells
Apoptosis 0,43%
Apoptosis+stress 0,05%
Live 99,51%
Stress 0,01%
Annexin V-H
MitoSox Red-H
CCCP / Main
live 53,17%
Q4-2 12,45%
block mito 12,03%
dead cells 22,34%
APC-H
7aad-H
2 / cells
Apoptosis 41,01%
Apoptosis+stress 5,57%
Live 53,24%
Stress 0,18%
3a / cells
Apoptosis 54,17%
Apoptosis+stress 2,25%
Live 43,52%
Stress 0,05%
3b / cells
Apoptosis 57,56%
Apoptosis+stress 2,44%
Live 39,95%
Stress 0,06%
4a / cells
Apoptosis 3,27%
Apoptosis+stress 5,93%
Live 88,55%
Stress 2,26%
4b / cells
Apoptosis 41,82%
Apoptosis+stress 9,91%
Live 47,31%
Stress 0,97%
5a / cells
Apoptosis 43,15%
Apoptosis+stress 24,54%
Live 31,78%
Stress 0,53%

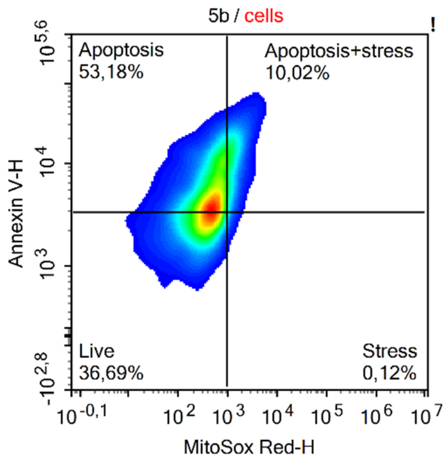
5b / cells
Apoptosis
53,18%
Apoptosis+stress
10,02%
Live
36,69%
Stress
0,12%
Annexin V-H
MitoSox Red-H
!

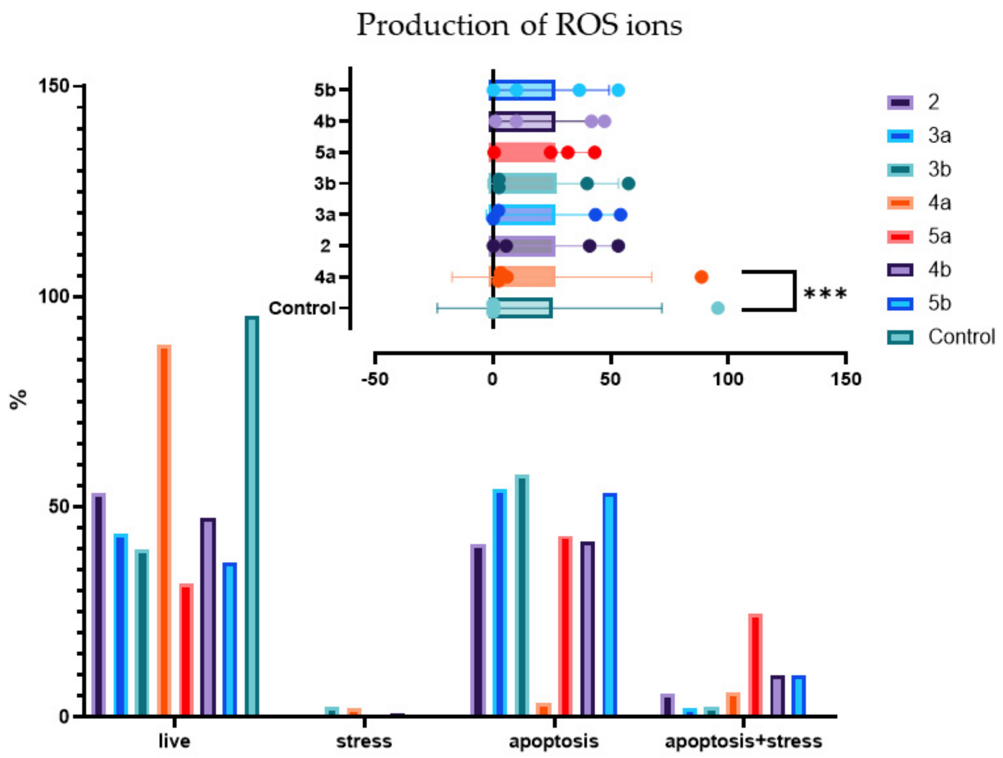

Figure S2. This study examines the impact of synthesized compounds on intracellular ROS ions levels. ΔΨm and drug concentrations in Jurkat cells were treated with hybrid molecules and ionic compounds at CC50 for four hours using flow cytometry. (A) The levels of ROS ions levels were evaluated through the use of MitoSOX™ Red staining, in accordance with the instructions provided by the manufacturer (catalog number M36008, Thermo Fisher Scientific Inc.) and Annexin. (B) A histogram of the detected cellular changes is provided below. The presence of triple asterisks (***) indicates that the observed difference is statistically significant at the $p < 0.001$ level.

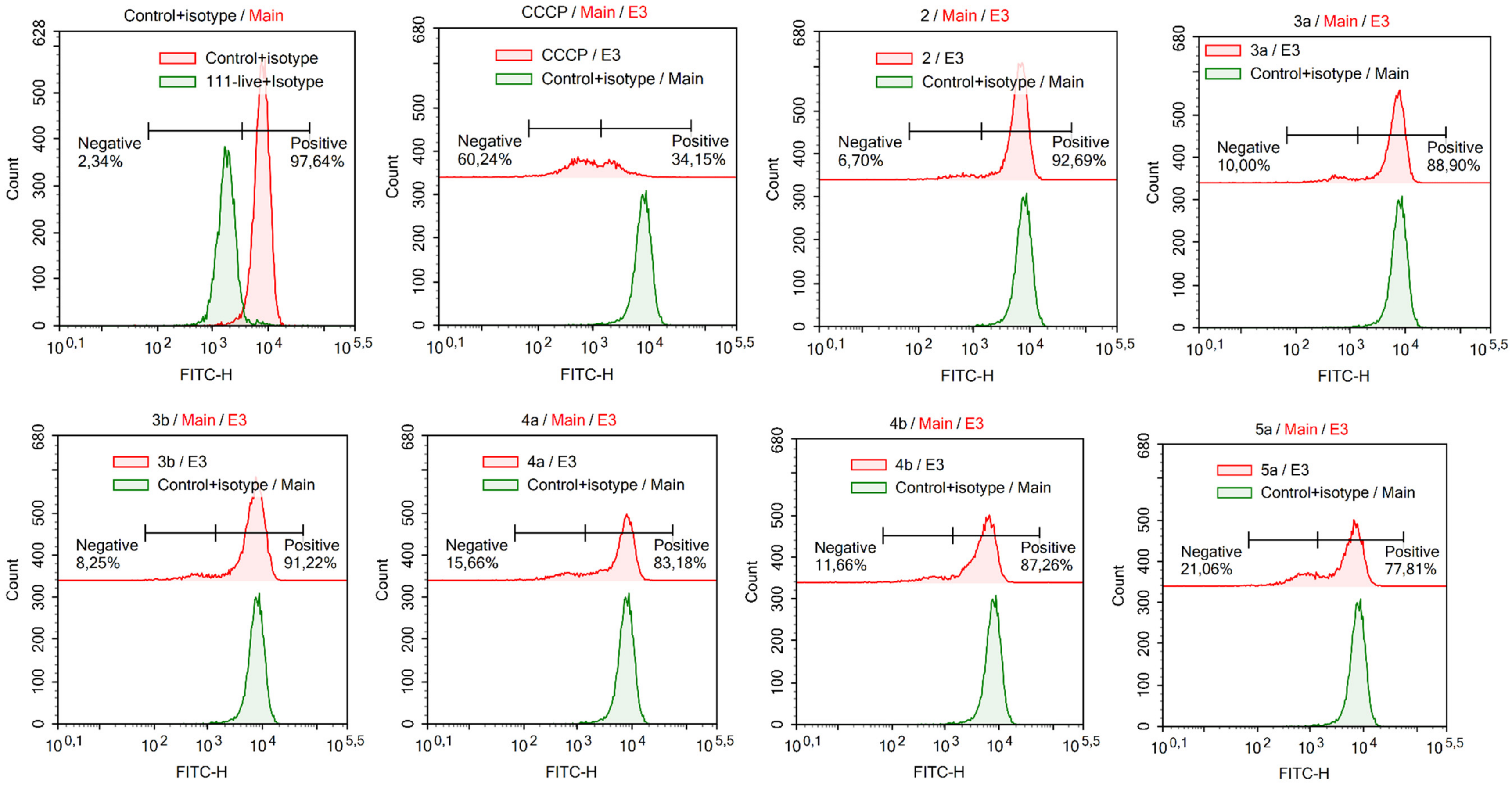
Control+isotype / Main
Control+isotype
111-live+Isotype
Negative 2,34%
Positive 97,64%
CCCP / Main / E3
CCCP / E3
Control+isotype / Main
Negative 60,24%
Positive 34,15%
2 / Main / E3
2 / E3
Control+isotype / Main
Negative 6,70%
Positive 92,69%
3a / Main / E3
3a / E3
Control+isotype / Main
Negative 10,00%
Positive 88,90%
3b / Main / E3
3b / E3
Control+isotype / Main
Negative 8,25%
Positive 91,22%
4a / Main / E3
4a / E3
Control+isotype / Main
Negative 15,66%
Positive 83,18%
4b / Main / E3
4b / E3
Control+isotype / Main
Negative 11,66%
Positive 87,26%
5a / Main / E3
5a / E3
Control+isotype / Main
Negative 21,06%
Positive 77,81%
Count
FITC-H

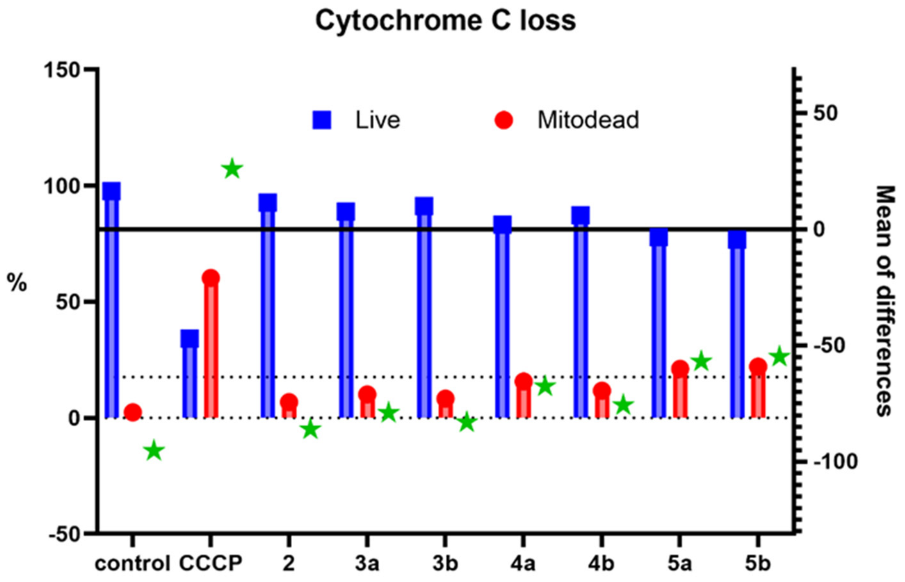

Figure S3. The figure depicts the detection of cytochrome c in Jurkat culture cells, which have been stained with the FlowCellect Cytochrome c Kit. Graph A illustrates the histogram of Jurkat cells treated with a combination of synthesized hybrid molecules and lithocholic acid-based ionic compounds at a CC50 concentration. The incubation period was four hours. Graph B illustrates the ratio of cells with damaged and undamaged mitochondria. The graphs illustrate a reduction in the fluorescence levels of cytochrome c for the Jurkat cell line. The control compound, carbonyl cyanide m-chlorophenylhydrazone (CCCP), was administered at a concentration corresponding to its IC50 value. The presence of green asterisks indicates that the observed difference is statistically significant at the $p < 0.001$ level.
